# Experimental and clinical progress of in utero hematopoietic cell transplantation therapy for congenital disorders

**DOI:** 10.3389/fphar.2022.851375

**Published:** 2022-09-02

**Authors:** Chunyu Shi, Lu Pan, Zheng Hu

**Affiliations:** ^1^ National-Local Joint Engineering Laboratory of Animal Models for Human Diseases, The First Hospital of Jilin University, Changchun, China; ^2^ Department of Gastrointestinal Colorectal and Anal Surgery, China-Japan Union Hospital of Jilin University, Changchun, China; ^3^ Department of Pediatric Immunology, Allergy and Rheumatology, The First Hospital of Jilin University, Changchun, China

**Keywords:** in utero transplantation, hematopoietic stem cell, chimerism, tolerance induction, inherent disorders

## Abstract

In utero hematopoietic cell transplantation (IUHCT) is considered a potentially efficient therapeutic approach with relatively few side effects, compared to adult hematopoietic cell transplantation, for various hematological genetic disorders. The principle of IUHCT has been extensively studied in rodent models and in some large animals with close evolutionary similarities to human beings. However, IUHCT has only been used to rebuild human T cell immunity in certain patients with inherent immunodeficiencies. This review will first summarize the animal models utilized for IUHCT investigations and describe the associated outcomes. Recent advances and potential barriers for successful IUHCT are discussed, followed by possible strategies to overcome these barriers experimentally. Lastly, we will outline the progress made towards utilizing IUHCT to treat inherent disorders for patients, list out associated limitations and propose feasible means to promote the efficacy of IUHCT clinically.

## 1 Introduction

Extensive progress has been made in the field of fetal interventions for congenital disorders. Since its first description in 1982 (Harrison et al., 1982), fetal surgeries have successfully treated various anatomical anomalies; however, they are limited to the correction of structural anomalies. For some congenital hematopoietic disorders, postnatal hematopoietic stem cell transplantation (HSCT) remains the only therapy; however, it is often restricted by limited histocompatible donors and severe treatment-associated morbidity and mortality. In recent years, remarkable advances in prenatal screening and molecular diagnostics have improved the possibility of diagnosing congenital disorders early during gestation. (Flake and Zanjani, 1999a). In utero hematopoietic cell transplantation is a non-myeloablative approach that can avoid the concerns of postnatal treatment and potentially cure various congenital disorders (Surbek et al., 2001). Compared to postnatal therapy, IUHCT allowing physicians to intervene in the treatment of diseases before birth. Furthermore, certain biological advantages unique to the fetal environment provide compelling reasons for favoring prenatal therapy over postnatal therapy (Vrecenak and Flake, 2013). However, the clinical success of IUHCT has been limited to severe combined immunodeficiency (SCID) over the last few decades (Vrecenak and Flake, 2013). Herein, we review the rationale, current progress, and potential clinical applications of IUHCT. We also discuss the barriers to engraftment and potential strategies to overcome them.

## 2 Rationale for in utero hematopoietic cell transplantation

The IUHCT notion is supported by the first report of a natural experiment studied by Owen in 1945, when he found that dizygotic cattle twins with shared placental circulation were chimeric postnatally (Owen, 1945). In 1953, Billingham showed that introduction of foreign antigen into early gestation fetuses resulted in the development of immunologic tolerance towards the foreign antigen (Billingham et al., 1953). The most compelling rationale for IUHCT is the induction of fetal specific immunologic tolerance to donor cells due to the naive immune system of the fetus. During the early gestation period, the fetal immune system undergoes a self-education process which occurs primarily in the fetal thymus. The outer region of thymus (cortex) supports the positive selection of T lineage progenitors whose TCRs react to self-major histocompatibility complex (MHC) molecules in a proper strength. The survival cells then travel to the inner thymus (medulla), where negative selection induces apoptosis of the progenitors that possess potential to response with self-antigens, including self-MHC, presented on thymic antigen presenting cells (APCs). The process results in deletion of self-reactive T cells and in a state of self-tolerance (central tolerance) (Palmer, 2003; Takahama, 2006). However, the thymic deletion is incomplete, self-reactive T cells that escape the deletion are suppressed by regulatory T cells (Tregs) mediated peripheral tolerance (Vrecenak and Flake, 2013). Central and peripheral tolerance leaves the fetus to recognize self-antigens and eliminate foreign antigens (Tai-MacArthur et al., 2021). Theoretically, introduction of allogenic cells by IUHCT ahead of the formation of functional fetal adaptive immune system could result in deletion of alloreactive T cells and induction of Treg cells, resulting in complete donor-specific immune tolerance (Vrecenak and Flake, 2013).

Another rationale for IUHCT is the potential hematopoietic niches available for donor cell homing and engraftment during the large-scale migration of hematopoietic stem cells (HSCs) during fetal development (Vrecenak and Flake, 2013). Hematopoiesis emerges from the yolk sac and aorto-gonadal-mesonephros regions. HSCs then circulate to the fetal liver, where HSCs undergo a dramatic expansion. Finally, HSCs colonize the bone marrow (BM), where hematopoiesis takes place throughout the adult life (Dzierzak and Speck, 2008; Bertrand et al., 2010; Gao et al., 2018). Moreover, the fetal environment also supports the expansion and differentiation of donor stem cells (Sagar et al., 2019). Manipulation of regular migration to selectively favor donor HSCs may help overcome competition from the host hematopoietic compartment and improve donor engraftment after IUHCT (Almeida-Porada et al., 2016). In addition, before and during the second trimester of gestation, the fetal BM is relatively empty, allowing donor cell engraftment without requiring myelosuppression, compared to postnatal HSCT (Surbek et al., 2001).

The third advantage of IUHCT is the small fetus size. At 12–13 weeks of gestation, when IUHCT is ideally performed, the human fetus weighs less than 50 g. Therefore, it is possible to deliver a much larger donor cell dosage on a fetal weight basis than could be provided postnatally.

## 3 Experimental models for in utero hematopoietic cell transplantation

IUHCT has been performed in many different animal models, of which murine models are most extensively used (Table 1). In the late 1970s, Fleischman and Mintz reported the first study of IUHCT (Fleischman and Mintz, 1979), in which an intraplacental injection of donor BM cells was administered at gestational day 11 (E11) into fetal mice with genetic anemia based on c-kit deficiency. The results showed complete substitution with donor erythroid cells in homozygous anemic mice. Later, studies conducted by Mintz and Blazar reported that even a single normal donor HSC was sufficient to engraft and reconstruct normal hematopoiesis in a c-kit-deficient mouse model (Mintz et al., 1984; Blazar et al., 1995a). Blazer also demonstrated only lymphoid reconstitution (split chimerism) in a severe combined immunodeficiency (SCID) mouse model in which donor lymphoid cells showed proliferative and survival advantages (Blazar et al., 1995b). In normal mice without any immune or stem cell deficiency, competitive pressure from the host prevented donor cell engraftment and the level of chimerism remained very low (Carrier et al., 1995; Kim et al., 1998; Donahue et al., 2001). These studies highlight the importance of host cell competition and the engraftment advantage achieved by immune deficiency. The low level of chimerism in normal mice resulted in donor-specific immune tolerance, which might form the basis of postnatal cell transplantation (Carrier et al., 1995; Kim et al., 1999). Further studies have shown that intravenous injection allows for the delivery of much higher cell numbers, resulting in increased chimerism (Peranteau et al., 2007; Boelig et al., 2016).

**TABLE 1:** IUT in murine model.

Ref (year)	Donor	Recepient	Injection site	Injection time	Number of source cell	Chimerism	Disease
Fleischman and Mintz (1979)	C57BL/6 or DBA/2	W/W or W^v^/W^v^	Intraplacental	E11	1 × 10^5^ FL (E13-E15)	In peripheral blood (PB) is more in W/W than in W^v^/W^v^	NO
Mintz et al. (1984)	C57BL/6 or BALB/c	W/W or W^f^/W^f^	Intraplacental	E11	1–2 × 10^5^ FL (E13)	In PB is more in W/W than in W^f^/W^f^	NO
Blazar et al. (1995a)	C57BL/6	W41/W41	IP	E13/14	1.5 × 10^6^ BM	In Multiple tissues (57%–80% T cells, 10%–15% B cells,27%–43% granulocytes	NO
Blazer et al. (1995b)	C57BL/6 or B10.BR	C57BL/6Sz-scid/scid	IP	E14/15	2 × 10^5^–2 × 10^6^ BM	100% T and B cell reconstituion	NO
Carrier et al. (1995)	C57BL/6	BALB/c or C57BL/6	IP, intraplacental	E11-E13	5 × 10^5^ FL (E15-E16)	0.0001% (spleen and liver)-0.6% (PB)	NO
Carrier et al. (1997)	C57BL/6	BALB/c or C57BL/6	IP, intraplacental	E11-E13	5 × 10^5^ BM, FL (E15-E16)	0.0003%–0.4% (liver and spleen), 0.002%–2.4% (PB)	NO
Archer et al. 1997)	C57BL/6	NOD/SCID	IP	E13.5	8 × 10^5^ lin-depleted BM	17%–55% (PB), 8%–26% (BM), and 20–68% (spleen)	NO
Blazer et al. (1998)	C57BL/6 or BALB/c	BALB/c-SCID	IP	E15/E16	1–4 × 10^6^ T cell-depleted BM, whole BM	High frequency engraftment in PB, BM, thymus, and spleen	NO
Kim et al. (1998)	C57BL/6	BALB/c	IP	E13-E16	1 × 10^6^ BM	Microchimerism (range<0.1%) in PB	NO
Turner et al. (2015)	Human	NOD/SCID	IP	E13/E14	6–8 × 10^5^ CD34^+^ cells (FBM, FL)	0·6%–0·9% (PB), 0.2%–15% (BM), 0·2%–3·4% (spleen)	NO
Kim et al. (1999)	DBA/2	BALB/c	IP	E14	1 × 10^6^ BM	Successful skin grafts in 2 of 3 mice	NO
Carrier et al. (2000)	C57BL/6	BALB/c	IP	E12/E13	8 × 10^4^–5 × 10^5^ BM derived C-kit + cells	Microchimerism (<0.01%) in PB, liver and spleen	NO
Donahue et al. (2001)	C57BL/6	BALB/c	IP	E11-E13	1–2 × 10^5^ Spleen derived Sca-1+Lin-, C-kit + Lin- cells	Microchimersim (<0.001%) in PB	NO
Chou et al. (2001)	C57BL/6	BALB/c	IP	E13-E15	1.5 × 10^6^ (BM), 2 × 10^5^ (BM derived CD80lowCD86−pDC)	Significant Higher engraftment in PB and 0.01%–4% (BM), 0.001%–1.21% (spleen) in BM + pDC group	NO
Casal and Wolfe (2001)	Mice transgenic for the human genomic GUSB DNA and mutant for murine GUSB (TG)	MPSVII	intraplacental	E13.5	1 × 10^5^ or 1 × 10^6^ FL (E13.5)	Low-level chimerism (<0.1%) in PB	Mucopolysaccharidosis type VII
Mackenzie et al. (2002)	Rosa26	Muscular dystrophy (MDX)	IP	E14	1 to 5 × 10^6^ BM, FL (E14)	0.2%–9% (PB)	Muscular dystrophy
Peranteau et al. (2002)	C57BL/6	BALB/c	IP	E13/E14	5 × 10^6^ T-cell depleted BM	2–6% (PB) improved to 80% with low-dose TBI + same-donor TCD BMT	NO
Hayashi et al. (2002)	C57BL/6	BALB/c	IP	E14/E15	5 × 10^6^ T-cell depleted BM	Blood macrochimerism (>3%) to nearly 100% with same donor lymphocyte infusion	NO
Waldschmidt et al. (2002)	C57BL/6 or BALB/c	BALB/c-SCID	IP	E15/E16	4 × 10^6^ T cell-depleted, whole BM	All B-cell subsets restores in PB and BM	NO
Taylor et al. (2002)	C57BL/6	BALB/c-SCID	IP	E15/E16	1 × 10^6^ T-cell depleted BM, FL	74% (PB in FL group) 11% (PB in BM group)	NO
Sefrioui et al. (2002)	C57BL/6	BALB/c	IP	E13	5 × 10^4^ cytokine-stimulated sca-1+lin- cells	Undectectable in PB and spleen	NO
Barker et al. (2003)	C57BL/6	MPSVII	IH	E14	—FL (E14/E15)	1.1%–8.7% (PB)	Mucopolycaccharidosis type VII
Chen et al. (2004)	B6D2F1 (C57BL/6 × DBA/2)	C57BL/6	IP	E13	1 × 10^6^ Undectectable in PB and spleen BM, T-cell depleted BM, T-cell depleted BM with CD8	Low-level chimerism (<0.2%) in PB, spleen and BM.	NO
Hayashi et al. (2004)	C57BL/6	BALB/C	IP	E14	5 × 10^6^ T-cell depleted BM, 0.25–1 × 10^6^ splenocytes from B6 mice presensitized to BALB/C alloantigen (pSPC)	Full chimerism in PB	NO
Schoeberlein et al. (2004)	C57BL/6 Human	NOD/SCID	IP	E13.5	1 × 10^5^ to 1 × 10^6^ Undectectable in PB and spleenFL (E13.5) or human CD34+	(Mouse FL group) 49.9% (PB), 5.2% (BM) and 86.2% (spleen) (Human CD34^+^ group) Undetected in PB	NO
Moustafa et al. (2004)	R1 embryonic stem (ES) cells, C57BL/6J	BALB/C	IP	E13.5	1 × 10^9^/kg BM, FL (E13.5)	Low-level chimerism in PB (<0.4%)	NO
Rio et al. (2005)	(B6.SJL-PtprcaPep3b/BoyJ × DBA/2J) F1	(C57BL/6J × DBA/2J) F1	IP	E14.5	5 × 10^6^ BM or 2 × 10^4^ Lin-Sca-1+	1.0%–6.2% (PB in BM group) 0.5%–35.5% (PB in Lin-Sca-1+ group)	NO
Frattini et al. (2005)	CMV/GFP CD-1 transgenic mice	oc−/−	IP	E14.5	5 × 10^6^ BM	Improved survival	Autosomal recessive osteopetrosis
Shaaban et al. (2006)	BALB/c	C57BL/6	IP	E14	—BM with or without vascular endothelial growth factor (VEGF) and stem cell factor (SCF)	0.01%–0.1% (PB)	NO
Peranteau et al. (2006)	C57BL/6TgN (act-EGFP) OsbY01 (B6GFP)	BALB/c or Swiss Webster	IV	E14	20 × 10^6^ BM or 1 × 10^5^ c-kit + sca-1+lin-	Significant higher-level chimerism with CD26 inhibition	NO
Peranteau et al. (2007)	B6GFP	BALB/c or C57BL/6	IV	E14	20 × 10^6^ BM	70% allogeneic recipient loss chimerism	NO
Durkin et al. (2008)	BALB/c	B6Ly5.2	IP	E14	2 × 10^4^—2 × 10^6^ FL (E14)	0.1%–10.5% (PB)	NO
Chen et al. (2008)	C57BL/6	FVB/N	IP	E14	1–10 × 10^6^ BM or T-cell depleted BM	0.25%–2.06% (PB)	NO
Guillot et al. (2008)	Human	oim/oim	IP	E13.5-E15	1 × 10^6^ fetal blood mesenchymal stem cells (MSCs)	More donor cells in bone tissues compared with other organs	Osteogenesis imperfecta
Tondelli et al. (2009)	CD-1 TG (ACTB-EGFP)	oc/oc	IH	E13.5	2 × 10^5^ FL (E12.5)	Improved survival	Autosomal recessive osteopetrosis
Panaroni et al. (2009)	CMV/GFP CD-1	BrtlIV mice	IH	E13.5/E14.5	5 × 10^6^ BM	Multiple tissues	Osteogenesis imperfecta (OI)
Merianos et al. (2009)	B6GFP	BALB/c	IV	E14	1 × 10^7^ BM	1.35% (PB), 0.6% (Spleen), 0.38% (BM)	NO
Chen et al. (2009b)	HS23-eGFP transgenic mice	Kun-Ming Bai	IP	E12.5, E13.5 or E14.5	5 × 10^4^ BM derived Sca-1+	1.55% (PB)	NO
Liuba et al. (2009)	C57/B16	X-SCID mice	IP	E14-E16	200 or 1,000 LMPPs (LSKCD34+FLT3^hi^), 200 HSC (LSKCD34- FLT3-)	33% or 53% (PB in LMPPs group) 43% (PB in HSC group)	NO
Chen et al. (2010)	C57BL/6	FVB/N	IP	E14	5–10 × 10^6^ T-cell depleted BM	0.01%–8.75% (PB), 0.04%–3.46% (multiple tissue)	NO
Troeger et al. (2010)	B6GFP	C57/BL6 or BALB/c	IP	E12 or E13.5	1 × 10^5^ FL (E14)	(E12) Microchimerism in maternal tissues	NO
Nijagal et al. (2011a)	NOD.CD45.1.uGFP	C57BL/6 × BALB/c (F1)	IH	E14.5	2.5 × 10^6^ FL (E13.5-E14.5)	Increased maternal cell chimerism in fetal PB	NO
Nijagal et al. (2013)	BALB/c	(B6 × TCR-TgB6.Thy1.1.4C) or (B6 ×B6.Thy1.1.TCR75) (F1)	IH	E14.5	2.5 × 10^6^ FL (E13.5-E14.5)	Equivalent chimerism in PB	NO
Chen et al. (2013)	C57BL/6	FVB/N	IP	E14	1–10 × 10^5^ splenic lymphocytes	Low-level chimerism (<0.1%) in PB, spleen and thymus	NO
Chen et al. (2015)	C57BL/6	FVB/N	IP	E14	5–10 × 10^6^ T-cell depleted BM	0.01%–10% (PB)	NO
Peranteau et al. (2015)	SJL/J	SCD and Thal mice	IP	E14	5 × 10^6^ T-cell depleted BM	1–4% (PB)	Sickle cell disease and β-thalassemia
Kim et al. (2016)	B6GFP	BALB/c	IP	E14	10×10^6^ BM	<10% (PB)	NO
Boelig et al. (2016)	B6GFP	C57BL/6	IV, IP, IH	E14	5 × 10^6^ BM	4%–6% (PB in IV group),2%–4% (PB in IP, IH group)	NO
Shangaris et al. (2018)	B6 (CD45.1) or BALB/cJ	C57BL/6J	IP	E13.5	1 × 10^4^ or 5 × 10^4^ amniotic fluid stem cells (AFSC) (E13.5)	5%–10% (PB), 5%–10% (BM), nearly 5% (spleen)	NO
Riley et al. (2018)	B6GFP	BALB/c	IV	E14	1 × 10^7^ BM	10%–20% (PB)	NO
Witt et al. (2018a)	Human	NSG	IH	E13.5/E14.5	2.5–5 × 10^4^ CB CD34^+^	1%–10% (PB)	NO
Borhani-Haghighi et al. (2019)	C57BL6J	C57BL6J	Lateral ventricle	E17	1 × 10^5^ Neural stem cells (NSCs)	Improved survival and injury	Prenatal white matter injury
Vrecenak et al. (2020)	B6GFP	BALB/c	IV	E14	10 × 10^6^ BM, 5 × 10^6^ BM derived Lin-, 1 × 10^5^ LSK	20%–30% (PB) 15%–20% (Liver, spleen, BM)	NO
Loukogeorga et al. (2019b)	B6GFP or BALB/c	C57BL/6J or BALB/c	IV	E14	1 × 10^4^ AFSC (E13)	19.2% (PB), 17.6% (BM), 17.9% (spleen), 6.4% (thymus)	NO
Riley et al. (2020)	B6GFP	BALB/c	IV	E20	9.7 × 10^6^ (T-cell depleted BM), 0.5 × 10^6^ CD4^+^CD25+splenocytes	1% (PB), 2.7% (BM), 9.9% (spleen)	NO
Nguyen et al. (2020)	CX3CR1-GFP	MPS7	IH	E13.5/E14.5	2.5–5 × 10^6^ FL (E14.5)	0.1%–35% (PB), 0.1%–25% (BM)	Mucopolysaccharidosis type VII
Shaw et al. (2021)	Human	SMA model mice	IP	E14	1 × 10^5^ AFSC	Higher engraftement in muscle and liver	Spinal muscular atrophy

Murine models are also used to study the mechanisms of donor-specific tolerance induction by IUHCT and various strategies to postnatally improve donor cell engraftment. Previous studies have shown that clonal deletion, anergy, and induction of donor-specific Tregs are essential for the induction of immune tolerance (Kim et al., 1999; Hayashi et al., 2004; Merianos et al., 2009; Nijagal et al., 2011a; Nijagal et al., 2011b). Further studies that administered low-dose total body irradiation (TBI) or busulfan to chimeric recipients followed by T-cell-depleted bone marrow transplantation (BMT) resulted in complete donor cell chimerism without the graft-versus-host disease (GVHD) (Peranteau et al., 2002; Ashizuka et al., 2006). Moreover, another strategy of using postnatal donor-specific lymphocyte infusion combined with IUHCT without BMT also resulted in complete donor cell chimerism without GVHD (Hayashi et al., 2002). These results highlight the crucial roles of host immune barrier and resident hematopoietic stem cell competition in compromising IUHCT efficacy, and implicate potential strategies to promote postnatal chimerism after successful induction of donor specific tolerance by IUHCT in clinical applications.

In addition to murine models, large animal models are also valuable and necessary preclinical tools for IUHCT study. The sheep model was the first large animal model to demonstrate sustained allogeneic engraftment after in utero transplantation of fetal stem cells (Flake et al., 1986) (Table 2). Moreover, *ex vivo* incubation of donor cells with growth factors enhanced engraftment of allogeneic stem cells (Zanjani et al., 1992a). Increasing the proportion of donor T cells also resulted in increased level of allogeneic chimerism, and approximately 1% donor T cells allowed significant engraftment without GVHD (Crombleholme et al., 1990). In sheep model, donor specific immune tolerance could also be induced by IUHCT, and postnatal injection of cells from the same donor enhanced the engraftment of donor cells (Zanjani, 1994). However, though 3%–5% stable chimerism was achieved, tolerance to renal transplantation was not observed in chimeric sheep (Hedrick et al., 1994). The sheep model also demonstrated persistent xenogeneic engraftment after transplantation of human HSCs (Zanjani et al., 1992b; Srour et al., 1992; Srour et al., 1993; Zanjani et al., 1994; Liechty et al., 2000; Noia et al., 2003; Noia et al., 2008; Tanaka et al., 2010; Goodrich et al., 2014; Jeanblanc et al., 2014). In addition, intracelomic transplantation of human CD34^+^ cells into fetal sheep resulted in more significant engraftment than that described in peritoneal transplantation (Noia et al., 2003). However, unlike the murine model results, the efficacies of intravenous and intraperitoneal transplantation were not significantly different in the sheep model (Tanaka et al., 2010). Moreover, cotransplation of stromal cell with HSC resulted in an increased level of chimerism in both xenogeneic and allogeneic sheep models (Almeida-Porada et al., 1999; Almeida-Porada et al., 2000).

**TABLE 2:** IUT in ovine model.

Ref (year)	Donor	Recepient	Injection site	Injection time	Number of source cell	Chimerism	Disease
Flake et al. (1986)	Sheep	Sheep	IP	45–65 gestational day	2–5 × 10^8^/kg FL (35–50 days of gestation)	14%–29% (PB)	NO
Crombleholme et al. (1990)	Sheep	Lamb	IP	90 gestational day	2 × 10^9^/kg T-cell depleted BM, BM	18% (PB in BM group), 6% (PB in T-cell depleted BM group)	NO
Zanjani et al. (1992a)	Sheep	Sheep	IP	48–54 gestational day	2 × 10^9^/kg FL (<60 days of gestation)	15%–25% (PB)	NO
Zanjani et al. (1992b)	Human	Sheep	IP	48–54 gestational day	2 × I0^9^−1 × 10^10^/kg FL (12–15 weeks of gestation)	0% (PB), 4%–9% (BM), 0–2% (liver)	NO
Srour et al. (1992)	Human	Sheep	IP	42–48 gestational day	2–4 × 10^4^ CD34+HLA-DR- BM	PB, 1.5% and 3.8% (BM)	NO
Srour et al. (1993)	Human	Sheep	IP	45–50 gestational day	4–10 × 10^4^ CD34+HLA-DR- BM	PB, 8.5%,11% < 0.1% (BM) chimerism	NO
Zanjani et al. (1993)	Sheep, Human	Sheep	IP	50 gestational day	1–2 × 10^9^/kg sheep FL (<60 days of gestation) 4–20 × 10^7^/kg human FL (12–14 weeks of gestation)	0%–3% (PB), 3%–6% (BM)	NO
Zanjani et al. (1994)	Human	Sheep	IP	58–49 gestational day	4.8 × 10^6^ CD45^+^ BM (previous chimeric sheep with human FL)	0.5%–3.2% (PB), 2.9%–8.8% (BM)	NO
Almeida-Porada et al. (1999)	Sheep	Sheep	IP	55–60 gestational day	3 × 10^6^ (T-cell depleted FL),1 × 10^7^ (T-cell depleted BM), 7.5 × 10^5^ (stromal cell)	4.3%–15.8% (PB), 9.8%–15.9% (BM)	NO
Almeida-Porada et al. (2000)	Human	Sheep	IP	55–60 gestational day	0.7–6.5 × 10^4^ (CD34^+^ BM), 5 × 10^4^–7.5 × 10^5^ (stromal cell)	18.9% (PB), 2% (BM)	NO
Liechty et al. (2000)	Human	Sheep	IP	65–85 gestational day	1–2 × 10^8^/kg MSCs	Multiple tissues	NO
Noia et al. (2003)	Human	Sheep	Intracelomic	40–45 gestational day	50 × 10^6^ (T-cell depleted), 1–2 × 10^5^ (CD34^+^)	Multiple tissues	NO
Noia et al. (2008)	Human	Sheep	Intracelomic	40–47 gestational day	10 × 10^4^–30 × 10^6^ CD34^+^ (BM or CB)	Multiple tissues	NO
Tanaka et al. (2010)	Human	Sheep	IV or IP	59–61 gestational day	1.4–6.3 × 10^5^ CD34^+^ (CB)	1.3% (PB)	NO
Abe et al. (2012)	Human	Sheep (Busulfan conditioned)	IH	45–49 gestational day	0.72–2.4 × 10^6^ CD34^+^ (CB)	1.1%–3.3% (BM)	NO
Goodrich et al. (2014)	Human	Sheep	IH	53–75 gestational day	1.0–1.8 × 10^6^ (MSC), 0.8–8 × 10^5^ (CD34^+^)	1.45%–22.37% (PB)	NO
Shaw et al. (2015)	Sheep	Sheep	IP	60–64 gestational day	2 × 10^4^ CD34^+^ AFSC, BM	1.6%–4.5% (PB)	NO
Jeanblanc et al. (2014)	Sheep, Human	Sheep	IP	45 or 65 gestational day	5 × 10^5^ (Sheep T-cell depleted BM), 1.4 × 10^6^ (Sheep CD34^+^ BM), 4 × 10^4^–5 × 10^5^ (human CD34^+^ BM)	3%–14% (PB in sheep donor group), 1%–3% (PB in human donor group)	NO
Mokhtari et al. (2016)	Sheep	Sheep	IP	60–65 gestational day	2.5 × 10^6^/kg (CD146+CXCL12 + VEGFR2-), 7.1 × 10^6^/kg (CD146+CXCL12 + VEGFR2+), 2.1 × 10^6^/kg (HSC)	15% (PB in 3 + group), 20% (BM in 3 + group)	NO

The canine model is an attractive large-animal model. The dog model demonstrated microchimerism after in utero transplantation of paternal dog BM or human HSCs (Omori et al., 1999; Blakemore et al., 2004) (Table 3). Moreover, IUHCT combined with postnatal same-donor boosting strategy using a low-dose busulfan conditioning regimen increased the level of chimerism from 1% to 35–45%, and cured leukocyte adhesion deficiency in a canine model (Peranteau et al., 2009). Vrecenak et al. (2014) demonstrated that intracardiac injection resulted in much higher levels of chimerism than intraperitoneal injection in normal canine models, without any conditioning or evidence of GVHD. Studies also showed that high doses of donor T cells with CD34^+^ resulted in microchimerism without GVHD (Petersen et al., 2007). More recently, Vrecenak et al. (2018) reported that a clear threshold of 1%–3% donor T cells allowed excellent engraftment without GVHD.

**TABLE 3:** IUT in canine model.

Ref (year)	Donor	Recepient	Injection site	Injection time	Number of source cell	Chimerism	Disease
Omori et al. (1999)	Human	Canine	Yolk sacs	37 gestational day	5 × 10^6^ BM with a reporter retroviral vector in long-term marrow cultures (LTMCs)	0.5%–5% (PB), 0.1%–1.3% (BM)	NO
Blakemore et al. (2004)	Paternal canine	Canine	IP	30–41 gestational day	1.3 × 10^8^–2.5 × 10^10^/kg CD34^+^ BM	Microchimerism (<1%) in multiple tissues	NO
Petersen et al. (2007)	Male canine	Canine	IP	35–38 gestational day	4.5 × 10^8^–1.3 × 10^9^/kg (CD34^+^ BM), 8 × 10^6^–8.8 × 10^8^/kg (T cells)	Microchimerism (0%–2%) in multiple tissues	NO
Peranteau et al. (2009)	Parental canine leukocyte adhesion deficiency (CLAD)	CLAD	IP	63 gestational day	1.7–4.8 × 10^8^/kg CD34^+^ BM	0.2%–1.6% (PB)	leukocyte adhesion deficiency
Vaags et al. (2011)	Canine	Canine	Yolk sacs	25 or 35 gestational day	1–5 × 10^6^ (MSC), 0.1–2.5 × 10^7^ (BM)	Detection of labeled cells in liver and BM	NO
Petersen et al. (2013)	Parental canine	Canine	IP	31–50 gestational day	0.09–3.4 × 10^9^/kg (CD34^+^ BM), 0.11–1.1 × 10^9^/kg (T cells)	0%–10% (multiple tissues)	NO
Vrecenak et al. (2014)	Maternal canine	Canine	IP or intracardiac (IC)	39–42.5 gestational day	2.5–4.1 × 10^8^/kg (CD3^+^ BM), 5.7 × 10^8^ to 1.7 × 10^9^/kg (CD34^+^ BM)	3%–39% (PB in IC group)	NO
Vrecenak et al. (2018)	Maternal canine	Canine	IC	38–43 gestational day	3.7 × 10^8^ to 2.7 × 10^9^/kg (CD3^+^ BM), 5.0 × 10^8^ to 5.8 × 10^9^/kg (CD34^+^ BM)	2%–40% (PB)	NO

Stable allogeneic and xenogeneic multilineage engraftment was also achieved in swine models (Lee et al., 2005b; Abellaneda et al., 2012; Fisher et al., 2013) (Table 4). IUHCT-induced chimerism in fetal swine resulted in donor-specific tolerance to renal and vascularized composite allograft transplantation without conditioning (Lee et al., 2005a; Mathes et al., 2005; Mathes et al., 2014). These studies support the possibility of using this strategy to cure fetuses that require postnatal organ transplantation.

**TABLE 4:** IUT in swine model.

Ref (year)	Donor	Recepient	Injection site	Injection time	Number of source cell	Chimerism	Disease
Rubin et al. (2001)	Swine	Swine	IV	50–55 gestational day	1.5 × 10^8^–1.5 × 10^9^ BM (whole), T-cell depleted BM	0.8%–0.95% (PB),1.1% (liver), 0.7% (spleen)	NO
Fujiki et al. (2003)	Human	Swine	IP	33–52 gestational day	1 × 10^7^–2.4 × 10^8^ (T-cell depleted CB), 3.9 × 10^5^–4 x 10^6^ (CD34^+^ CB)	Microchimerism (<1%) in PB and BM	NO
Lee et al. (2005b)	Swine	Swine	IV	50–55 gestational day	5 × 10^8^ T-cell depleted BM	Multiple tissues	NO
Lee et al. (2005a)	Swine	Swine	IV	50–55 gestational day	5 × 10^8^ T-cell depleted BM	Microchimerism in PB	NO
Mathes et al. (2005)	Swine	Swine	IV	50–55 gestational day	5 × 10^8^ T-cell depleted BM	0.16%–1.6% (PB)	NO
Abellaneda et al. (2012)	Human	Swine	IP	50 gestational day	2–15 × 10^6^ MNC (CB), MSC (BM)	Microchimerism in PB	NO
Fisher et al. (2013)	Human	Swine	IH	40 gestational day	1 × 10^7^ hepatocytes	Human albumin production	NO
Mathes et al. (2014)	Swine	Swine	IV	50–55 gestational day	5 × 10^8^ T-cell depleted BM	1.8%–90% (PB), multiple tissues	NO
Navarro Alvarez et al. (2015)	Baboon	GalT-KO Swine	IV	65 gestational day	18.5 × 10^6^ T-cell depleted BM	No detactable chimerism	NO

Finally, low levels of allogeneic chimerism were also achieved in non-human primates (Michejda et al., 1992; Asano et al., 2003; Shields et al., 2003; Mahieu-Caputo et al., 2004; Shields et al., 2004; Shields et al., 2005) (Table 5). Fetal immune suppression resulted in an increased level of chimerism, but the level remained low (Shields et al., 2004). Moreover, the level of long-term chimerism was not improved with postnatal donor cell infusion (Shields et al., 2004). The non-human primate model also allowed multilineage engraftment of human HSCs (Tarantal et al., 2000). In addition to these large animal models, chimerism was also observed in rats (Chen et al., 2009a; Li et al., 2012; Lazow et al., 2021), rabbits (Wengler et al., 2005; Martínez-González et al., 2012; Moreno et al., 2012), and cats (Abkowitz et al., 2009). These studies in mice and large-animal models established the foundation for clinical application of IUHCT.

**TABLE 5:** IUT in primate model.

Ref (year)	Donor	Recepient	Injection site	Injection time	Number of source cell	Chimerism	Disease
Michejda et al. (1992)	Primate	Primate	IP	118, 120, and 125 gestational day	5 × 10^7^/kg BM	PB chimerism	NO
Tarantal et al. (2000)	Human	Primate	IP	50–56 gestational day	5 × 10^6^ CD34^+^ PBMC (with or without T cells)	0.1%–1.7% (BM)	NO
Shields et al. (2003)	Primate	Primate	IP	0.34–0.38 gestation	9.9 × 10^8^–4.4 × 10^9^/kg (CD34^+^ BM), 2.6 × 10^5^–1.1 × 10^8^/kg (T cells)	0.4%–10.7% (PB), 0.1–16.8% (BM)	NO
Asano et al. (2003)	Primate	Primate	IP or IV	49–61 gestational day	3.6–4.8 × 10^6^ embryonic stem cells	Multiple tissues	NO
Shields et al. (2004)	Primate	Primate	IP	0.34–0.38 gestation	2.6–5.2 × 10^9^/kg CD34^+^ BM	Microchimerism (<1.0%) in PB and BM	NO
Mahieu-Caputo et al. (2004)	Primate	Primate	IH	89, 90, and 120 gestational day	12–30 × 10^6^ fetal hepatocytes (89–120 days of gestation)	Donor Hepatocyte chimerism	NO
Shields et al. (2005)	Primate	Primate	IP	0.34–0.38 gestation	1.18–5.2 × 10^9^/kg CD34^+^ BM or PB	Microchimerism (<1%) in PB and BM	NO

## 4 Barriers to in utero hematopoietic cell transplantation

**FIGURE 1:**
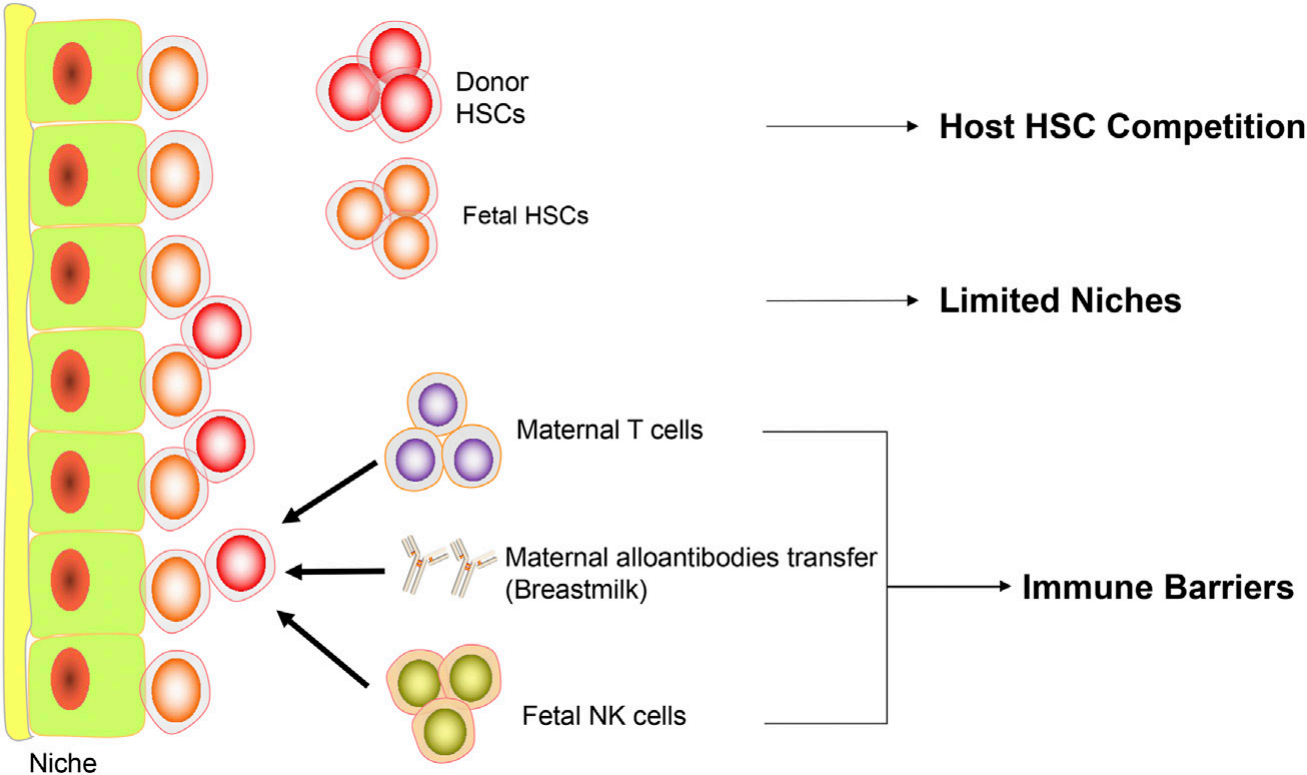
Barriers to IUHCT. Diagram representing the current barriers to IUHCT. Firstly, in normal recipients, the fetal HSCs compete with donor HSCs. Secondly, there are limited niches available in the fetal microenvironment. Thirdly, maternal T cells, maternal alloantibodies transferred through breastmilk and fetal NK cells consist of immune barriers.

Despite the compelling rationale of IUHCT, poor engraftment in most animal models suggests the existence of significant barriers to successful engraftment after IUHCT (Figure 1).

### 4.1 Host cell competition

In the c-kit-deficient mouse model, in which the donor cells have a competitive advantage, a single normal donor HSC is sufficient to engraft and reconstitute normal hematopoiesis (Mintz et al., 1984). Moreover, though the number of donor HSCs in the BM remains relatively low, nearly complete lymphoid lineage reconstitution is achieved in SCID mouse models and X-SCID patients (Blazar et al., 1995b; Flake et al., 1996), in which donor lymphoid cells have a proliferative and survival advantage. These studies have demonstrated the effectiveness of this competitive advantage in the setting of a proliferative defect in one or more lineages. In contrast, in normal mouse models, donor BM cells migrate to the fetal liver rapidly after IUHCT, followed by a rapid decrease of engraftment level, demonstrating host fetal cells outcompete donor BM cells (Shaaban et al., 1999). Because the host fetal HSCs have a distinct competitive advantage over donor adult HSCs due to their rapid cycling and expansion kinetics (Jordan et al., 1995; Leung et al., 1999; Shaaban et al., 1999; Rosler et al., 2000). Peranteau et al. (2007) reported how this competition limits long-term donor cell engraftment by transplantation of massive doses of BM cells (2 × 10^11^ cells/kg) in a congenic mouse model, which resulted in long-term donor cell engraftment levels below 10%.

### 4.2 Limited niches within the host

Although the fetal microenvironment is fundamentally different from any postnatal system, a relatively valid comparable model is the postnatal nonmyeloablative syngeneic mouse model. In this mouse model, similar to syngeneic IUHCT, the host hematopoietic compartment is intact and the donor and recipient cells are genetically equal. Studies in this model have demonstrated a dose-dependent increase in donor cell engraftment with repetitive large doses of syngeneic donor cells (Stewart et al., 1993; Rao et al., 1997). A further study in this model demonstrated that administration of the donor cells over several separate infusions, rather than in one injection, did not increase engraftment levels (Ramshaw et al., 1995). Recently, Shimoto et al. (2017) demonstrated that transplantation of a vast number of HSCs (up to 390% of the total number of endogenous HSCs) into unconditioned mice resulted in a two-fold increase in the total number of HSCs (endogenous plus transplanted). Strikingly, they found that these donor cells did not compete with host HSCs, but engrafted distinct niches. These collective studies convincingly show that there are a large number of empty niches in normal BM, and donor HSCs can engraft into the BM without competing with host HSCs (Ding, 2017).

During fetal development, since the large migration of HSCs from yolk sac and aorto-gonadal-mesonephros regions to BM, there is an associated rapid expansion of the hematopoietic niches for the homing and engraftment of circulating HSCs. It is reasonable to suppose the niches available in prenatal microenvironment might exceed the niches in the postnatal environment (Flake and Zanjani, 1999a).

However, in the fetal sheep model, increasing the allogeneic and xenogeneic donor cell doses (10^6^ to 10^10^ cells/kg) results in an eventual plateau of engraftment efficiency, and a further increase in donor cells does not affect the donor engraftment (Zanjani et al., 1997). Thus, the available studies illustrate that there is not an abundance of niches available in the fetal microenvironment compared with those in the postnatal BM microenvironment.

In adult SCID mice, selective depletion of host HSCs with a c-kit antibody (ACK2) before BM transplantation results in engraftment levels of up to 90%. However, the chimerism level is only about 0.1%–1% in unconditioned recipients (Czechowicz et al., 2007). A recent study indicated that in utero depletion of host HSCs with ACK2 before neonatal congenic hematopoietic cell transplantation leads to higher engraftment levels (Derderian et al., 2014). These collective studies suggest that vacating host HSC niches may lead to high engraftment levels after IUHCT.

### 4.3 Immunological barriers

Peranteau et al. (2007) demonstrated that after transplantation of high doses of allogeneic or congenic BM cells into fetal mice, only 30% of allogeneic recipients sustained long-term chimerism, whereas 100% of congenic recipients remained chimeric. Recently, studies by Shangaris et al. (2018) and Loukogeorgakis et al. (2019b) showed that 100% of congenic recipients remained macrochimerism compared to 29% or 0% of allogeneic recipients with microchimerism after in utero transplantation of amniotic fluid stem cells. These results strongly suggest the engraftment advantage of congenic stem cells over allogeneic stem cells and the existence of immune barriers resulting in the elimination of allogeneic cells after IUHCT.

In 2008, Merianos et al. (2009) first demonstrated the existence of a maternal immune barrier after IUHCT, and an adaptive alloimmune response was induced by the transfer of maternal antibodies to pups *via* breast milk, resulting in the loss of chimerism in allogeneic recipients. In this study, chimerism in allogenic recipients remained at 100% when the pups were fostered by a naive mother. The most important observation of this study was that in the absence of the maternal immune response, the recipients were tolerant of allogeneic donor cells *via* partial deletion of donor-specific T cells and the induction of Tregs.

A subsequent study by Nijagal et al. (2011a) demonstrated that maternal leukocytes increased significantly in murine fetuses after IUHCT. More importantly, donor engraftment improved dramatically in the fetuses of T cell-deficient mothers, indicating that the maternal T cells limited donor engraftment. Furthermore, when the donor cells were matched to the mother, there was no difference in engraftment between the syngeneic and allogeneic fetal recipients.

Recently, Riley et al. (2018) demonstrated that after in utero transplantation of donor HSCs in a novel murine model, in which donor-specific antibodies were already present at the time of injection, the maternal donor-specific IgG was transferred to the fetus in utero and caused rapid rejection of allogeneic donor cells, resulting in a mean engraftment level of 0%. These collective studies suggest that the maternal immune response may be a significant barrier to the success of engraftment after IUHCT in some murine models. Whether maternal immunization is a limitation for engraftment in large animal models and clinical applications requires further investigation. Despite unsolved issues, we hypothesize that it may be prudent to use donor cells either from the mother or matched to the mother’s stem cells to avoid maternal immunization and improve engraftment in IUHCT for the treatment of many congenital diseases.

In addition to the maternal immune barrier, early studies also support the existence of a fetal immune barrier to IUHCT. Clinical success has been observed in the treatment of x-SCID patients with paternally derived stem cells (Touraine et al., 1989; Flake et al., 1996; Wengler et al., 1996), and failure in the treatment of sickle cell disease and thalassemia (Orlandi et al., 1996; Westgren et al., 1996). The maternal immune response in these cases was intact regardless of their clinical outcome, but the effect of maternal immune barrier was not apparent.

Some previous studies suggested a significant role for NK cells in immune rejection (Carrier et al., 2000; Donahue et al., 2001), and also suggested that NK cells may pose the earliest barrier to engraftment following allogeneic IUHCT (Alhajjat et al., 2010; Alhajjat et al., 2015). Recently, Durkin et al. (2008) proved that engraftment or rejection after IUHCT correlated with the level of initial chimerism. All mice exhibiting >1.8% chimerism demonstrated allogeneic NK cell tolerance; however, mice with <1.8% chimerism underwent NK cell-mediated rejection. Furthermore, rejection did not occur when NK host cells were depleted from mice with <1.8% chimerism but reoccurred when NK cells were allowed to recover. The same team also demonstrated that depletion of fetal but not maternal NK cells enables stable engraftment of allogeneic cells following IUHCT and donor-to-host MHC transfer (trogocytosis) as an intrinsic mechanism regulating the development and maintenance of NK cell tolerance in prenatal chimeras (Alhajjat et al., 2013; Alhajjat et al., 2015). Although these data still need to be studied in large animal models, they illustrated the importance of NK cells in the fetus for clinical IUHCT success.

## 5 Strategies to overcome engraftment barriers

**FIGURE 2:**
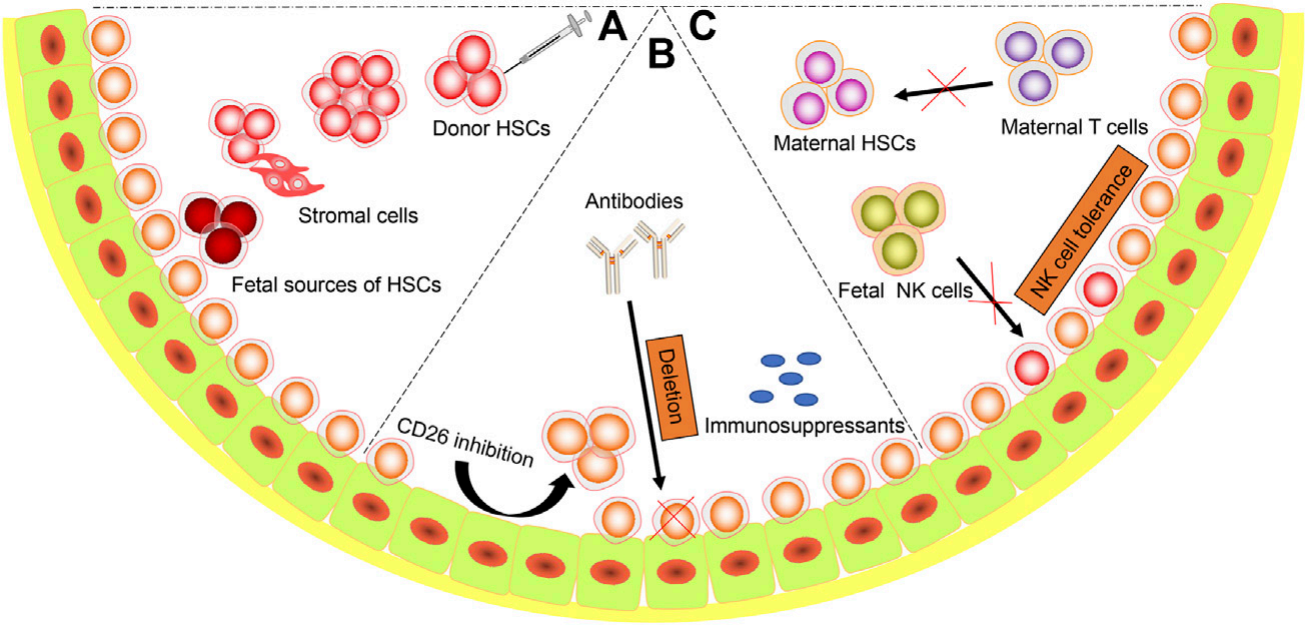
Strategies to Succeed IUHCT. Diagram representing the possible strategies to overcome the barriers. **(A)** Host cell competition. a. Proper routes (intravenous, intraperitoneal, intrahepatic, intracelomic and intracardiac) and time of administration; b. Increasing cell doses. c. Cotransplantation with stromal cells. d. Using fetal sources of stem cells (fetal liver, amniotic fluid stem cells, etc.); **(B)** Limited niches. a. Mobilize HSCs from niches. b. Antibodies targeted to host HSCs. c. Maternal immunosuppressants administration; **(C)** Immune barriers. a. Matched maternal HSCs. b. Induction of host NK cells tolerance.

During the last few decades, efforts have been made to overcome the barriers to successful IUHCT in normal recipients and improve the engraftment of donor cells without GVHD. These strategies fall into three categories: 1) providing competitive advantage for donor cells, 2) increasing receptive niches for donor cells, 3) overcoming immune barriers (Figure 2).

### 5.1 Providing competitive advantage for donor cells

#### 5.1.1 Using fetal sources of stem cells

Hematopoietic stem cells can be isolated from fetal liver, umbilical cord blood, and adult BM. Taylor et al. (2002) and Harrison et al. (1997) demonstrated that fetal liver cells had a higher competitive engraftment advantage than adult BM in allogeneic SCID mouse models and xenogeneic sheep models. In addition to these sources, many studies demonstrated the hematopoietic characteristics of amniotic fluid stem cells (AFSCs) and achieved long-term engraftment with AFSC after IUHCT in congenic mouse and autologous sheep model (Shaw et al., 2015; Shangaris et al., 2018; Loukogeorgakis et al., 2019b). But whether AFSCs can engraft in allogeneic or xenogeneic animal models needs further studies. Due to the fetal origin of AFSCs, long-term engraftment was also significantly higher than that achieved with BM (Loukogeorgakis et al., 2019b). These studies suggest that fetal donor cells are more competitive to some extent.

#### 5.1.2 Proper routes and time of injection of donor cells

Administration of donor cells can be accomplished through intravenous (IV), intraperitoneal (IP), intrahepatic (IH), intracelomic and intracardiac routes. Compared with IP and IH route, intravenous administration of donor cells resulted in higher levels of chimerism in murine models (Boelig et al., 2016). However, no difference was detected in sheep models (Tanaka et al., 2010). In addition, intracelomic and intracardiac injection of donor cells resulted in higher level of chimerism than that achieved in intraperitoneal injection in sheep and canine models. (Noia et al., 2003; Vrecenak et al., 2014). It is still a question which route is the most suitable. Prior to clinical application, more studies of injection routes need to be evaluated in the nonhuman primate model.

The injection time is crucial, especially for non-immunodeficient fetuses. As mentioned previously, the “window of opportunity” has been a major concern for the experimental and clinical success of IUHCT. The evidence suggests that although the transplant can occur outside the window of opportunity, significant modifications to the technique or the conditions of the transplant are needed to achieve a successful transplant. If donor cells were introduced in the early gestation period when the immune system of the host was naïve, the foreign antigen could be recognized as “self” and not rejected. In the murine model, most of the studies have been performed at E13.5 or E14.5 (Table 1). Chen et al. (2009b) performed IUHCT at E12.5, resulting in the highest chimerism rate compared to those at E13.5 and E14.5; however, the average engraftment was not high (1.55%). In the canine model, studies proved that the initial thymic selection started at around 40 days of gestation and obtained maximal engraftment at 42 days of gestation (Petersen et al., 2013; Vrecenak et al., 2014). In the sheep model, Jeanblanc et al. (2014) found between 45 and 51 days of gestation, the osteoblastic/endosteal niche started developing, and their number increased with gestational age. Moreover, significantly higher engraftment was observed at 65 days of gestation, indicating that a fully functional BM microenvironment improved engraftment. In humans, this period correlates with events that occur from 12 to 14 weeks of gestation (Darrasse-Jeze et al., 2005; Alhajjat et al., 2010). Further studies are needed to evaluate the appropriate time for IUHCT in humans.

#### 5.1.3 Increasing donor cell dosage or repopulating competency

Studies from sheep model showed that engraftment after IUHCT was dose-dependent, which seemed to reach a plateau above the optimal dose (Zanjani et al., 1997). Moreover, Vrecenak et al. (2020) demonstrated that increasing doses of Lin-cells combined with BM could dramatically improve both allogeneic early and late engraftment after IUHCT.

Recently, Loukogeorgakis et al. (2019a) performed IUHCT of HSCs decorated with glycogen synthase kinase-3 (GSK3) inhibitor-loaded nanoparticles, which enhanced the repopulating capacity of donor cells and dramatically improved long-term allogeneic engraftment. Shaaban et al. (2006) demonstrated that pre-incubation of donor cells with vascular endothelial growth factor (VEGF) and stem cell factor (SCF) resulted in improved short-term chimerism. Moreover, Goodrich et al. (2014) and Almeida-Porada et al. (2000) proved that cotransplantation BM with stromal cells could also improve allogeneic and xenogeneic engraftment (Almeida-Porada et al., 1999).

### 5.2 Increasing receptive niches for donor cells

#### 5.2.1 Mobilize hematopoietic stem cells from the host niches

Peranteau et al. (2006) found that *ex vivo* inhibition of CD26 in donor cells increased donor cell homing to the fetal liver and increased short- and long-term allogeneic engraftment. The CXCR4|SDF-1α and α4β1|VCAM-1 pathways are critical for HSC recruitment into the BM after postnatal transplantation. Kim et al. (2016) observed that maternal administration of AMD3100 (a CXCR4 antagonist) and firategrast (an α4β1 antagonist) prior to IUHCT would mobilize host HSCs from fetal liver and increase long-term allogeneic engraftment significantly in a mouse model. Goodrich et al. (2004) performed fetal cotransplantation with AMD3100 and human CD34^+^ cells in sheep model resulted in improved chimerism.

#### 5.2.2 Maternal immunosuppressants administration

Studies in sheep models showed that maternal administration of busulfan before IUHCT increased engraftment of donor cells (Abe et al., 2012). Similarly, Shields et al. (2004) proved that fetal administration of corticosteroids and antithymocyte globulin (ATG) before IUHCT is associated with an increase in the level of progenitor and BM chimerism in nonhuman primate models. However, whether these immunosuppressants can be safely used for clinical application needs further studies.

#### 5.2.3 Administration of antibodies targeted to host hematopoietic stem cells

Derderian et al. (2014) proved that in utero depletion of host HSCs with a c-Kit receptor antibody led to significantly increased engraftment after neonatal congenic hematopoietic cell transplantation. Chhabra et al. (2016) and Witt et al. (2018b) demonstrated that preconditioning with an anti-c-Kit antibody and CD47 antagonist markedly improved long-term engraftment in both prenatal and postnatal mouse models. Palchaudhuri et al. (2016) showed that conditioning with CD45 blockage enable long-term donor cell engraftment (>90%) in immunocompetent mice and complete correction of a sickle-cell anemia model. A recent study showed that administration of anti-human CD117 antibody resulted in the depletion of host HSCs and the improvement in donor cell engraftment in nonhuman primates and humanized NSG mice (Kwon et al., 2019). The above mentioned studies support the notion that depleting host HSC niches through “silver bullet” may be a viable method of improving donor cell chimerism after IUHCT, though further studies are needed in more prenatal animal models.

### 5.3 Overcoming immune barriers

With a better understanding of the maternal and fetal immune barriers, some promising strategies may be applied to improve the engraftment of donor cells. Firstly, donor cells harvested from the mother or matched to the mother’s stem cells are most likely to be used in initial clinical trials. Aiming for an initial chimerism threshold of >1.8% with host NK cell tolerance may improve engraftment of donor cells (Durkin et al., 2008). Deleting NK cells within the fetus before IUHCT may also facilitate engraftment of donor cells (Durkin et al., 2008). However, further studies are required in large animal models. The absolute number and concentration of donor T cells also dramatically affects engraftment. This strategy focuses on inducing a graft-versus-hematopoietic effect with the use of donor T cells, but without GVHD. Previous studies in murine, sheep, canine, swine, and non-human primate models emphasized the importance of T cells and demonstrated that approximately 1%–2% donor T cells are felicitous in facilitating donor cell engraftment without GVHD (Crombleholme et al., 1990; Shields et al., 2003; Lee et al., 2005b; Chen et al., 2008; Vrecenak et al., 2018). In addition, using donor T cells presensitized to the recipient with donor HSCs can also provide an engraftment advantage to donor cells without GVHD (Bhattacharyya et al., 2002; Hayashi et al., 2004). A recent study also showed that in utero injection of BM with regulatory T cells, either from chimeric mice or from naive donors, could promote allogeneic engraftment in late-gestation mouse models (Riley et al., 2020). However, further studies are needed to understand the role of T cells and Tregs in large animal models before their clinical application.

## 6 Clinical application of in utero hematopoietic cell transplantation

Since the first clinical application of IUHCT in a bare lymphocyte syndrome fetus (Touraine et al., 1989), there have been approximately 50 reported cases of IUHCT during the past 30 years, targeting various diseases with different donor cell sources and transplantation protocols. Unfortunately, the success of this process has been limited mainly to fetuses with SCID (Flake et al., 1996; Wengler et al., 1996; Westgren et al., 2002). However, in successful IUHCT cases, the patients manifested engraftment only in the T-cell lineage (split chimerism), similar to postnatal results in HSCT treatment (Buckley et al., 1999). Thus far, there is little evidence demonstrating the advantages of prenatal treatment over postnatal treatment in patients with X-linked SCID (Flake and Zanjani, 1999b). As for other immunodeficiency disorders, such as chronic granulomatous disease and Chediak-Higashi syndrome, no detectable engraftment was achieved in the treated children (Cowan and Golbus, 1994; Flake and Zanjani, 1997; Muench et al., 2001).

Similarly, the use of IUHCT to treat hemoglobinopathies has mostly been unsuccessful. There have been 12 attempts to treat β-thalassemia with IUHCT, and only two have showed detectable postnatal engraftment (Slavin et al., 1992; Orlandi et al., 1996; Westgren et al., 1996; Sanna et al., 1999; Vrecenak and Flake, 2013). In α-thalassemia, three attempts at IUHCT were made, where only one demonstrated microchimerism and donor-specific immune tolerance (Cowan and Golbus, 1994; Westgren et al., 1996; Hayward et al., 1998). Currently, Horvei et al. (2021) are performing a phase I clinical trial (NCT02986698) of IUHCT using maternal cells as donors for treatment of α-thalassemia. As discussed previously, the presence of maternal cells in the fetus result in fetal tolerance, transplantation of maternal cells provides the highest likelihood of success. If the one-step protocol results in low levels of chimerism, a combined strategy would be performed with postnatal maternal cells transplantation.

In sickle cell anemia, fetal liver cells were injected to a female fetus at 13 weeks of gestation, however, engraftment was not detected at 3 months after her birth (Westgren et al., 1996). In another two unpublished clinic cases that applied IUHCT for sickle cell anemia therapy also failed in donor cell engraftment (Westgren, 2006). Metabolic storage diseases, another type of inherited disorder, may also benefit from IUHCT. Seven attempts were made and two cases showed engraftment (Bambach et al., 1997; Flake and Zanjani, 1997; Touraine et al., 1997; Touraine et al., 2004); however, one patient showed no improvement, while the other died prenatally, probably due to GVHD. For other genetic disorders, such as osteogenesis imperfecta, two attempts resulted in microchimerism and transient clinical effects (Le Blanc et al., 2005; Götherström et al., 2014). To date, the results of clinical cases demonstrated that clinical IUHCT, along with current methods, is not able to establish therapeutic levels of engraftment in recipients without significant immunodeficiency. Because of this, only a few recent clinical attempts at IUHCT have been reported. These clinical cases used various donor cell sources and IUHCT was performed at different times during gestation. These inconsistencies have made it impossible to determine the specific factors responsible for the low donor engraftment or failure in clinical practice. Therefore, it is vital to perform more controlled IUHCT in animal models to optimize the protocols before clinical application. In 2015, MacKenzie et al. (2015) produced an international consensus statement describing guidelines for IUSCT clinical trials.

Currently, two strategies can be used in clinical applications. The first is to perform IUHCT alone to achieve therapeutic engraftment levels; however, such a strategy might only be possible for diseases that require very low levels of engraftment for therapeutic success. The most favorable target disease for this approach is X-linked SCID, and other diseases, such as chronic granulomatous disease, hyper-IgM syndrome, and leukocyte adhesion deficiency, which are characterized by an SCID phenotype, may benefit from such a strategy. However, further studies on such diseases are required to optimize the current strategy. For diseases requiring high levels of engraftment for therapeutic success, such as hemoglobinopathies, the most compelling strategy is performing IUHCT to induce donor-specific tolerance, followed by postnatal minimal or non-toxic conditioning of HSCTs from the same donor to increase donor engraftment. As mentioned before, this strategy significantly reduces the initial chimerism required for clinical success to 1%–2%. From the studies of murine, sheep, and canine models discussed earlier (Zanjani, 1994; Hayashi et al., 2002; Peranteau et al., 2002; Ashizuka et al., 2006; Peranteau et al., 2009), on the basis of the initial chimerism after IUHCT, donor engraftment can be enhanced dramatically to complete or near-complete levels. More importantly, this strategy lowers the chimerism threshold, which may be required for clinical success.

Recently, Peranteau et al. (2015) showed that IUHCT combined with the same donor non-myeloablative allogeneic BM transplants corrected the disease phenotype in mice with β-thalassemia and sickle cell disease. Moreover, IUHCT-induced donor-specific tolerance may allow for postnatal organ transplantation without immunosuppression. Chen et al. (2010) demonstrated that the success of postnatal donor skin transplantation was dependent on the level of donor cell chimerism after IUHCT in murine models. Studies in swine and canine models support the combined strategy for successful postnatal renal transplants from the same donor without immunosuppression (Lee et al., 2005a; Mathes et al., 2005; Vrecenak et al., 2014). These results highlight the therapeutic potential of IUHCT combined with postnatal same-donor “boosting” transplantation in the clinical setting.

## 7 Conclusion and perspectives

IUHCT has great potential for the treatment of numerous congenital hematological, genetic, and immunological disorders. However, this therapy has so far only been successfully achieved in fetuses with SCID. There are many hurdles remaining for IUHCT to overcome before it can be used as a therapeutic alternative for specific diseases. Challenges for IUHCT are mainly related to overcoming the competitive barriers to engraftment in the fetus and to better understand the maternal and fetal immune barriers to engraftment in large animal models and humans. Studies in murine and large animal models suggest that, under limited circumstances, a single IUHCT may result in high levels of donor cell engraftment to ameliorate the target disease. However, it seems unlikely to reach a therapeutic level of engraftment without the development of safe myeloablative drugs or options in the human fetus. Hopefully, through a greater understanding of induction and maintenance of immune tolerance and stem cell biology, new innovative methods and diverse source of donor cells can be applied to achieve high donor chimerism. Although limited clinical studies at the current time, the strategy of prenatal tolerance induction followed by postnatal HSC transplantation from the same donor to enhance engraftment is promising in the future. Further insights into the fetal hematopoietic ontogeny will further facilitate the improvement of therapeutic strategies based on IUHCT in the future.

## References

[B1] Abe T. Masuda S. Tanaka Y. Nitta S. Kitano Y. Hayashi S.fnm (2012). Maternal administration of busulfan before in utero transplantation of human hematopoietic stem cells enhances engraftments in sheep. Exp. Hematol. 40(6), 436–444. 10.1016/j.exphem.2012.01.018 22306296

[B2] Abellaneda J. M. Ramis G. Martínez-Alarcón L. Majado M. J. Quereda J. J. Herrero-Medrano J. M. (2012). Generation of human-to-pig chimerism to induce tolerance through transcutaneous in utero injection of cord blood-derived mononuclear cells or human bone marrow mesenchymals cells in a preclinical program of liver xenotransplantation: Preliminary results. Transpl. Proc. 44 (6), 1574–1578. 10.1016/j.transproceed.2012.05.016 22841218

[B3] Abkowitz J. L. Sabo K. M. Yang Z. Vite C. H. Shields L. E. Haskins M. E. (2009). In utero transplantation of monocytic cells in cats with alpha-mannosidosis. Transplantation 88 (3), 323–329. 10.1097/TP.0b013e3181b0d264 19667933 PMC2742773

[B4] Alhajjat A. M. Durkin E. T. Shaaban A. F. (2010). Regulation of the earliest immune response to in utero hematopoietic cellular transplantation. Chimerism 1 (2), 61–63. 10.4161/chim.1.2.13147 21327049 PMC3023625

[B5] Alhajjat A. M. Lee A. E. Strong B. S. Shaaban A. F. (2015). NK cell tolerance as the final endorsement of prenatal tolerance after in utero hematopoietic cellular transplantation. Front. Pharmacol. 6, 51. 10.3389/fphar.2015.00051 25852555 PMC4364176

[B6] Alhajjat A. M. Strong B. S. Durkin E. T. Turner L. E. Wadhwani R. K. Midura E. F. (2013). Trogocytosis as a mechanistic link between chimerism and prenatal tolerance. Chimerism 4 (4), 126–131. 10.4161/chim.26666 24121538 PMC3921193

[B7] Almeida-Porada G. Atala A. Porada C. D. (2016). In utero stem cell transplantation and gene therapy: Rationale, history, and recent advances toward clinical application. Mol. Ther. Methods Clin. Dev. 5, 16020. 10.1038/mtm.2016.20 27069953 PMC4813605

[B8] Almeida-Porada G. Flake A. W. Glimp H. A. Zanjani E. D. (1999). Cotransplantation of stroma results in enhancement of engraftment and early expression of donor hematopoietic stem cells in utero. Exp. Hematol. 27 (10), 1569–1575. 10.1016/s0301-472x(99)00090-9 10517499

[B9] Almeida-Porada G. Porada C. D. Tran N. Zanjani E. D. (2000). Cotransplantation of human stromal cell progenitors into preimmune fetal sheep results in early appearance of human donor cells in circulation and boosts cell levels in bone marrow at later time points after transplantation. Blood 95 (11), 3620–3627. 10.1182/blood.v95.11.3620 10828053

[B10] Archer D. R. Turner C. W. Yeager A. M. Fleming W. H. (1997). Sustained multilineage engraftment of allogeneic hematopoietic stem cells in NOD/SCID mice after in utero transplantation. Blood 90 (8), 3222. 10.1182/blood.v90.8.3222 9376606

[B11] Asano T. Ageyama N. Takeuchi K. Momoeda M. Kitano Y. Sasaki K. (2003). Engraftment and tumor formation after allogeneic in utero transplantation of primate embryonic stem cells. Transplantation 76 (7), 1061–1067. 10.1097/01.Tp.0000090342.85649.81 14557753

[B12] Ashizuka S. Peranteau W. H. Hayashi S. Flake A. W. (2006). Busulfan-conditioned bone marrow transplantation results in high-level allogeneic chimerism in mice made tolerant by in utero hematopoietic cell transplantation. Exp. Hematol. 34 (3), 359–368. 10.1016/j.exphem.2005.11.011 16543070 PMC1934419

[B13] Bambach B. J. Moser H. W. Blakemore K. Corson V. L. Griffin C. A. Noga S. J. (1997). Engraftment following in utero bone marrow transplantation for globoid cell leukodystrophy. Bone Marrow Transpl. 19 (4), 399–402. 10.1038/sj.bmt.1700665 9051254

[B14] Barker J. E. Schuldt A. J. Lessard M. D. Jude C. D. Vogler C. A. Soper B. W. (2003). Donor cell replacement in mice transplanted in utero is limited by immune-independent mechanisms. Blood Cells Mol. Dis. 31 (2), 291–297. 10.1016/s1079-9796(03)00134-7 12972038

[B15] Bertrand J. Y. Chi N. C. Santoso B. Teng S. Stainier D. Y. Traver D. (2010). Haematopoietic stem cells derive directly from aortic endothelium during development. Nature 464 (7285), 108–111. 10.1038/nature08738 20154733 PMC2858358

[B16] Bhattacharyya S. Chawla A. Smith K. Zhou Y. Talib S. Wardwell B. (2002). Multilineage engraftment with minimal graft-versus-host disease following in utero transplantation of S-59 psoralen/ultraviolet a light-treated, sensitized T cells and adult T cell-depleted bone marrow in fetal mice. J. Immunol. 169 (11), 6133–6140. 10.4049/jimmunol.169.11.6133 12444116

[B17] Billingham R. E. Brent L. Medawar P. B. (1953). Actively acquired tolerance of foreign cells. Nature 172 (4379), 603–606. 10.1038/172603a0 13099277

[B18] Blakemore K. Hattenburg C. Stetten G. Berg K. South S. Murphy K. (2004). In utero hematopoietic stem cell transplantation with haploidentical donor adult bone marrow in a canine model. Am. J. Obstet. Gynecol. 190 (4), 960–973. 10.1016/j.ajog.2004.01.014 15118622

[B19] Blazar B. R. Taylor P. A. Mcelmurry R. Tian L. Vallera D. A. (1998). Engraftment of severe combined immune deficient mice receiving allogeneic bone marrow via in utero or postnatal transfer. Blood 92 (10), 3949–3959. 10.1182/blood.v92.10.3949 9808589

[B20] Blazar B. R. Taylor P. A. Vallera D. A. (1995a). Adult bone marrow-derived pluripotent hematopoietic stem cells are engraftable when transferred in utero into moderately anemic fetal recipients. Blood 85 (3), 833–841. 10.1182/blood.v85.3.833.bloodjournal853833 7833485

[B21] Blazar B. R. Taylor P. A. Vallera D. A. (1995b). In utero transfer of adult bone marrow cells into recipients with severe combined immunodeficiency disorder yields lymphoid progeny with T- and B-cell functional capabilities. Blood 86 (11), 4353–4366. 10.1182/blood.v86.11.4353.bloodjournal86114353 7492797

[B22] Boelig M. M. Kim A. G. Stratigis J. D. McClain L. E. Li H. Flake A. W. (2016). The intravenous route of injection optimizes engraftment and survival in the murine model of in utero hematopoietic cell transplantation. Biol. Blood Marrow Transpl. 22 (6), 991–999. 10.1016/j.bbmt.2016.01.017 26797401

[B23] Borhani-Haghighi M. Mohamadi Y. Kashani I. R. (2019). In utero transplantation of neural stem cells ameliorates maternal inflammation-induced prenatal white matter injury. J. Cell Biochem. 120 (8), 12785–12795. 10.1002/jcb.28548 30861185

[B24] Buckley R. H. Schiff S. E. Schiff R. I. Markert L. Williams L. W. Roberts J. L. (1999). Hematopoietic stem-cell transplantation for the treatment of severe combined immunodeficiency. N. Engl. J. Med. 340 (7), 508–516. 10.1056/nejm199902183400703 10021471

[B25] Carrier E. Gilpin E. Lee T. H. Busch M. P. Zanetti M. (2000). Microchimerism does not induce tolerance afte*r* in utero *t*ransplantation and may lead to the development of alloreactivity. J. Lab. Clin. Med. 136 (3), 224–235. 10.1067/mlc.2000.108942 10985501

[B26] Carrier E. Lee T. H. Busch M. P. Cowan M. J. (1995). Induction of tolerance in nondefective mice after in utero transplantation of major histocompatibility complex-mismatched fetal hematopoietic stem cells. Blood 86 (12), 4681–4690. 10.1182/blood.v86.12.4681.bloodjournal86124681 8541562

[B27] Carrier E. Lee T. H. Busch M. P. Cowan M. J. (1997). Recruitment of engrafted donor cells postnatally into the blood with cytokines after in utero transplantation in mice. Transplantation 64 (4), 627–633. 10.1097/00007890-199708270-00014 9293877

[B28] Casal M. L. Wolfe J. H. (2001). In utero transplantation of fetal liver cells in the mucopolysaccharidosis type VII mouse results in low-level chimerism, but overexpression of beta-glucuronidase can delay onset of clinical signs. Blood 97 (6), 1625–1634. 10.1182/blood.v97.6.1625 11238101

[B29] Chen C. P. Liu S. H. Huang J. P. Aplin J. D. Wu Y. H. Chen P. C. (2009a). Engraftment potential of human placenta-derived mesenchymal stem cells after in utero transplantation in rats. Hum. Reprod. 24 (1), 154–165. 10.1093/humrep/den356 18845668

[B30] Chen J. C. Chang M. L. Huang S. F. Chang P. Y. Muench M. O. Fu R. H. (2008). Prenatal tolerance induction: Relationship between cell dose, marrow T-cells, chimerism, and tolerance. Cell Transpl. 17 (5), 495–506. 10.3727/096368908785095971 18714669

[B31] Chen J. C. Chang M. L. Lee H. Muench M. O. (2004). Haploidentical donor T cells fail to facilitate engraftment but lessen the immune response of host T cells in murine fetal transplantation. Br. J. Haematol. 126 (3), 377–384. 10.1111/j.1365-2141.2004.05040.x 15257710

[B32] Chen J. C. Kuo M. L. Ou L. S. Chang P. Y. Muench M. O. Shen C. R. (2010). Characterization of tolerance induction through prenatal marrow transplantation: The requirement for a threshold level of chimerism to establish rather than maintain postnatal skin tolerance. Cell Transpl. 19 (12), 1609–1622. 10.3727/096368910x516583 20719075

[B33] Chen J. C. Ou L. S. Yu H. Y. Chang H. L. Chang P. Y. Kuo M. L. (2013). Allogeneic lymphocytes exerted graft-versus-host rather than tolerogenic effects on preimmune fetuses. J. Surg. Res. 183 (1), 405–411. 10.1016/j.jss.2012.12.015 23295194

[B34] Chen J. C. Ou L. S. Yu H. Y. Kuo M. L. Chang P. Y. Chang H. L. (2015). Postnatal donor lymphocytes enhance prenatally-created chimerism at the risk of graft-versus-host disease. Am. J. Transl. Res. 7 (5), 941–949. 26175855 PMC4494145

[B35] Chen X. Gong X. L. Katsumata M. Zeng Y. T. Huang S. Z. Zeng F. (2009b). Hematopoietic stem cell engraftment by early-stage in utero transplantation in a mouse model. Exp. Mol. Pathol. 87 (3), 173–177. 10.1016/j.yexmp.2009.07.009 19666020

[B36] Chhabra A. Ring A. M. Weiskopf K. Schnorr P. J. Gordon S. Le A. C. (2016). Hematopoietic stem cell transplantation in immunocompetent hosts without radiation or chemotherapy. Sci. Transl. Med. 8 (351), 351ra105. 10.1126/scitranslmed.aae0501 PMC6668627 27510901

[B37] Chou S. H. Chawla A., . Lee T. H. Zhou Y., . Busch M. P. Balassanian R. (2001). Increased engraftment and GVHD after in utero transplantation of MHC-mismatched bone marrow cells and CD80low, CD86(-) dendritic cells in a fetal mouse model. Transplantation 72 (11), 1768–1776. 10.1097/00007890-200112150-00010 11740386

[B38] Cowan M. J. Golbus M. (1994). In utero hematopoietic stem cell transplants for inherited diseases. Am. J. Pediatr. Hematol. Oncol. 16 (1), 35–42. 7906103

[B39] Crombleholme T. M. Harrison M. R. Zanjani E. D. (1990). In utero transplantation of hematopoietic stem cells in sheep: The role of T cells in engraftment and graft-versus-host disease. J. Pediatr. Surg. 25 (8), 885–892. 10.1016/0022-3468(90)90197-h 1976135

[B40] Czechowicz A. Kraft D. Weissman I. L. Bhattacharya D. (2007). Efficient transplantation via antibody-based clearance of hematopoietic stem cell niches. Science 318 (5854), 1296–1299. 10.1126/science.1149726 18033883 PMC2527021

[B41] Darrasse-Jeze G. Marodon G. Salomon B. L. Catala M. Klatzmann D. (2005). Ontogeny of CD4+ CD25+ regulatory/suppressor T cells in human fetuses. Blood 105 (12), 4715–4721. 10.1182/blood-2004-10-4051 15731180

[B42] Derderian S. C. Togarrati P. P. King C. Moradi P. W. Reynaud D. Czechowicz A. (2014). In utero depletion of fetal hematopoietic stem cells improves engraftment after neonatal transplantation in mice. Blood 124 (6), 973–980. 10.1182/blood-2014-02-550327 24879814 PMC4126335

[B43] Ding L. (2017). HSC niche: Ample room for every guest stem cell. Blood, J. Am. Soc. Hematol. 129 (15), 2042–2043. 10.1182/blood-2017-02-765586 PMC5391626 28408418

[B44] Donahue J. Gilpin E. Lee T. H. Busch M. P. Croft M. Carrier E. (2001). Microchimerism does not induce tolerance and sustains immunity after in utero transplantation. Transplantation 71 (3), 359–368. 10.1097/00007890-200102150-00004 11233894

[B45] Durkin E. T. Jones K. A. Rajesh D. Shaaban A. F. (2008). Early chimerism threshold predicts sustained engraftment and NK-cell tolerance in prenatal allogeneic chimeras. Blood 112 (13), 5245–5253. 10.1182/blood-2007-12-128116 18796629 PMC2597617

[B46] Dzierzak E. Speck N. A. (2008). Of lineage and legacy: The development of mammalian hematopoietic stem cells. Nat. Immunol. 9 (2), 129–136. 10.1038/ni1560 18204427 PMC2696344

[B47] Fisher J. E. Lillegard J. B. McKenzie T. J. Rodysill B. R. Wettstein P. J. Nyberg S. L. (2013). In utero transplanted human hepatocytes allow postnatal engraftment of human hepatocytes in pigs. Liver Transpl. 19 (3), 328–335. 10.1002/lt.23598 23280879 PMC3600116

[B48] Flake A. W. Harrison M. R. Adzick N. S. Zanjani E. D. (1986). Transplantation of fetal hematopoietic stem cells in utero: The creation of hematopoietic chimeras. Science 233 (4765), 776–778. 10.1126/science.2874611 2874611

[B49] Flake A. W. Roncarolo M. G. Puck J. M. Almeida-Porada G. Evans M. I. Johnson M. P. (1996). Treatment of X-linked severe combined immunodeficiency by in utero transplantation of paternal bone marrow. N. Engl. J. Med. 335 (24), 1806–1810. 10.1056/nejm199612123352404 8943162

[B50] Flake A. W. Zanjani E. D. (1999a). In utero hematopoietic stem cell transplantation: Ontogenic opportunities and biologic barriers. Blood 94 (7), 2179–2191. 10.1182/blood.V94.7.2179.419k43_2179_2191 10498587

[B51] Flake A. W. Zanjani E. D. (1997). In utero hematopoietic stem cell transplantation. A status report. Jama 278 (11), 932–937. 10.1001/jama.1997.03550110070039 9302247

[B52] Flake A. W. Zanjani E. D. (1999b). Treatment of severe combined immunodeficiency. N. Engl. J. Med. 341 (4), 291–292. 10.1056/nejm199907223410416 10419393

[B53] Fleischman R. A. Mintz B. (1979). Prevention of genetic anemias in mice by microinjection of normal hematopoietic stem cells into the fetal placenta. Proc. Natl. Acad. Sci. 76 (11), 5736–5740. 10.1073/pnas.76.11.5736 42904 PMC411725

[B54] Frattini A. Blair H. C. Sacco M. G. Cerisoli F. Faggioli F. Catò E. M. (2005). Rescue of ATPa3-deficient murine malignant osteopetrosis by hematopoietic stem cell transplantation in utero. Proc. Natl. Acad. Sci. U. S. A. 102 (41), 14629–14634. 10.1073/pnas.0507637102 16195375 PMC1253616

[B55] Fujiki Y. Fukawa K. Kameyama K. Kudo O. Onodera M. Nakamura Y. (2003). Successful multilineage engraftment of human cord blood cells in pigs after in utero transplantation. Transplantation 75 (7), 916–922. 10.1097/01.Tp.0000057243.12110.7c 12698074

[B56] Gao X. Xu C. Asada N. Frenette P. S. (2018). The hematopoietic stem cell niche: From embryo to adult. Development 145 (2). 10.1242/dev.139691 PMC5825844 29358215

[B57] Goodrich A. D. Varain N. M. Jeanblanc C. M. Colon D. M. Kim J. Zanjani E. D. (2014). Influence of a dual-injection regimen, plerixafor and CXCR4 on in utero hematopoietic stem cell transplantation and engraftment with use of the sheep model. Cytotherapy 16 (9), 1280–1293. 10.1016/j.jcyt.2014.05.025 25108653 PMC4131210

[B58] Götherström C. Westgren M. Shaw S. W. Aström E. Biswas A. Byers P. H. (2014). Pre- and postnatal transplantation of fetal mesenchymal stem cells in osteogenesis imperfecta: A two-center experience. Stem Cells Transl. Med. 3 (2), 255–264. 10.5966/sctm.2013-0090 24342908 PMC3925052

[B59] Guillot P. V. Abass O. Bassett J. H. Shefelbine S. J. Bou-Gharios G. Chan J. (2008). Intrauterine transplantation of human fetal mesenchymal stem cells from first-trimester blood repairs bone and reduces fractures in osteogenesis imperfecta mice. Blood 111 (3), 1717–1725. 10.1182/blood-2007-08-105809 17967940

[B60] Harrison D. E. Zhong R. K. Jordan C. T. Lemischka I. R. Astle C. M. (1997). Relative to adult marrow, fetal liver repopulates nearly five times more effectively long-term than short-term. Exp. Hematol. 25 (4), 293–297. 9131003

[B61] Harrison M. R. Golbus M. S. Filly R. A. Callen P. W. Katz M. de Lorimier A. A. (1982). Fetal surgery for congenital hydronephrosis. N. Engl. J. Med. 306 (10), 591–593. 10.1056/nejm198203113061006 7057815

[B62] Hayashi S. Hsieh M. Peranteau W. H. Ashizuka S. Flake A. W. (2004). Complete allogeneic hematopoietic chimerism achieved by in utero hematopoietic cell transplantation and cotransplantation of LLME-treated, MHC-sensitized donor lymphocytes. Exp. Hematol. 32 (3), 290–299. 10.1016/j.exphem.2003.12.008 15003315

[B63] Hayashi S. Peranteau W. H. Shaaban A. F. Flake A. W. (2002). Complete allogeneic hematopoietic chimerism achieved by a combined strategy of in utero hematopoietic stem cell transplantation and postnatal donor lymphocyte infusion. Blood 100 (3), 804–812. 10.1182/blood-2002-01-0016 12130490

[B64] Hayward A. Ambruso D. Battaglia F. Donlon T. Eddelman K. Giller R. (1998). Microchimerism and tolerance following intrauterine transplantation and transfusion for alpha-thalassemia-1. Fetal Diagn Ther. 13 (1), 8–14. 10.1159/000020793 9605609

[B65] Hedrick M. H. Rice H. E. MacGillivray T. E. Bealer J. F. Zanjani E. D. Flake A. W. (1994). Hematopoietic chimerism achieved by in utero hematopoietic stem cell injection does not induce donor-specific tolerance for renal allografts in sheep. Transplantation 58 (1), 110–111. 7913560

[B66] Horvei P. MacKenzie T. Kharbanda S. (2021). Advances in the management of α-thalassemia major: Reasons to be optimistic. Hematology 2021 (1), 592–599. 10.1182/hematology.2021000295 34889445 PMC8791144

[B67] Jeanblanc C. Goodrich A. D. Colletti E. Mokhtari S. Porada C. D. Zanjani E. D. (2014). Temporal definition of haematopoietic stem cell niches in a large animal model of in utero stem cell transplantation. Br. J. Haematol. 166 (2), 268–278. 10.1111/bjh.12870 24673111 PMC4079736

[B68] Jordan C. T. Astle C. M. Zawadzki J. Mackarehtschian K. Lemischka I. R. Harrison D. E. (1995). Long-term repopulating abilities of enriched fetal liver stem cells measured by competitive repopulation. Exp. Hematol. 23 (9), 1011–1015. 7635180

[B69] Kim A. G. Vrecenak J. D. Boelig M. M. Eissenberg L. Rettig M. P. Riley J. S. (2016). Enhanced in utero allogeneic engraftment in mice after mobilizing fetal HSCs by α4β1/7 inhibition. Blood 128 (20), 2457–2461. 10.1182/blood-2016-06-723981 27650329 PMC5114489

[B70] Kim H. B. Shaaban A. F. Milner R. Fichter C. Flake A. W. (1999). In utero bone marrow transplantation induces donor-specific tolerance by a combination of clonal deletion and clonal anergy. J. Pediatr. Surg. 34 (5), 726–729. discussion 729-730. 10.1016/s0022-3468(99)90364-0 10359172

[B71] Kim H. B. Shaaban A. F. Yang E. Y. Liechty K. W. Flake A. W. (1998). Microchimerism and tolerance afterin UteroBone marrow transplantation in mice. J. Surg. Res. 77 (1), 1–5. 10.1006/jsre.1997.5255 9698523

[B72] Kwon H. S. Logan A. C. Chhabra A. Pang W. W. Czechowicz A. Tate K. (2019). Anti-human CD117 antibody-mediated bone marrow niche clearance in nonhuman primates and humanized NSG mice. Blood 133 (19), 2104–2108. 10.1182/blood-2018-06-853879 30617195 PMC6509543

[B73] Lazow S. P. Kycia I. Labuz D. F. Zurakowski D. Fauza D. O. (2021). Fetal hematogenous routing of a donor hematopoietic stem cell line in a healthy syngeneic model of transamniotic stem cell therapy. J. Pediatr. Surg. 56 (6), 1233–1236. 10.1016/j.jpedsurg.2021.02.035 33771370

[B74] Le Blanc K. Götherström C. Ringdén O. Hassan M. McMahon R. Horwitz E. (2005). Fetal mesenchymal stem-cell engraftment in bone after in utero transplantation in a patient with severe osteogenesis imperfecta. Transplantation 79 (11), 1607–1614. 10.1097/01.tp.0000159029.48678.93 15940052

[B75] Lee P. W. Cina R. A. Randolph M. A. Arellano R. Goodrich J. Rowland H. (2005a). In utero bone marrow transplantation induces kidney allograft tolerance across a full major histocompatibility complex barrier in Swine. Transplantation 79 (9), 1084–1090. 10.1097/01.tp.0000161247.61727.67 15880048

[B76] Lee P. W. Cina R. A. Randolph M. A. Goodrich J. Rowland H. Arellano R. (2005b). Stable multilineage chimerism across full MHC barriers without graft-versus-host disease following in utero bone marrow transplantation in pigs. Exp. Hematol. 33 (3), 371–379. 10.1016/j.exphem.2004.12.002 15730861

[B77] Leung W. Ramírez M. Civin C. I. (1999). Quantity and quality of engrafting cells in cord blood and autologous mobilized peripheral blood. Biol. Blood Marrow Transpl. 5 (2), 69–76. 10.1053/bbmt.1999.v5.pm10371358 10371358

[B78] Li H. Gao F. Ma L. Jiang J. Miao J. Jiang M. (2012). Therapeutic potential of in utero mesenchymal stem cell (MSCs) transplantation in rat foetuses with spina bifida aperta. J. Cell Mol. Med. 16 (7), 1606–1617. 10.1111/j.1582-4934.2011.01470.x 22004004 PMC3823228

[B79] Liechty K. W. MacKenzie T. C. Shaaban A. F. Radu A. Moseley A. M. Deans R. (2000). Human mesenchymal stem cells engraft and demonstrate site-specific differentiation after in utero transplantation in sheep. Nat. Med. 6 (11), 1282–1286. 10.1038/81395 11062543

[B80] Liuba K. Pronk C. J. Stott S. R. Jacobsen S. E. (2009). Polyclonal T-cell reconstitution of X-SCID recipients after in utero transplantation of lymphoid-primed multipotent progenitors. Blood 113 (19), 4790–4798. 10.1182/blood-2007-12-129056 19074736

[B81] Loukogeorgakis S. P. Fachin C. G. Dias A. Li H. Tang L. Kim A. G. (2019a). Donor cell engineering with GSK3 inhibitor-loaded nanoparticles enhances engraftment after in utero transplantation. Blood 134 (22), 1983–1995. 10.1182/blood.2019001037 31570489

[B82] Loukogeorgakis S. P. Shangaris P. Bertin E. Franzin C. Piccoli M. Pozzobon M. (2019b). Utero transplantation of expanded autologous amniotic fluid stem cells results in long-term hematopoietic engraftment. Stem Cells 37 (9), 1176–1188. 10.1002/stem.3039 31116895 PMC6773206

[B83] MacKenzie T. C. David A. L. Flake A. W. Almeida-Porada G. (2015). Consensus statement from the first international conference for in utero stem cell transplantation and gene therapy. Front. Pharmacol. 6, 15. 10.3389/fphar.2015.00015 25713535 PMC4322602

[B84] Mackenzie T. C. Shaaban A. F. Radu A. Flake A. W. (2002). Engraftment of bone marrow and fetal liver cells after in utero transplantation in MDX mice. J. Pediatr. Surg. 37 (7), 1058–1064. 10.1053/jpsu.2002.33844 12077771

[B85] Mahieu-Caputo D. Loux N. Simon L. Allain J. E. Beaudoin S. Bargy F. (2004). In utero allotransplantation of fetal hepatocytes in primates. Fetal Diagn Ther. 19 (1), 92–99. 10.1159/000074269 14646427

[B86] Martínez-González I. Moreno R. Petriz J. Gratacós E. Aran J. M. (2012). Engraftment potential of adipose tissue-derived human mesenchymal stem cells after transplantation in the fetal rabbit. Stem Cells Dev. 21 (18), 3270–3277. 10.1089/scd.2012.0032 22738094 PMC3516421

[B87] Mathes D. W. Solari M. G. Gazelle G. S. Butler P. E. Wu A. Nazzal A. (2014). Stable mixed hematopoietic chimerism permits tolerance of vascularized composite allografts across a full major histocompatibility mismatch in swine. Transpl. Int. 27 (10), 1086–1096. 10.1111/tri.12380 24963743

[B88] Mathes D. W. Solari M. G. Randolph M. A. Gazelle G. S. Yamada K. Huang C. A. (2005). Long-term acceptance of renal allografts following prenatal inoculation with adult bone marrow. Transplantation 80 (9), 1300–1308. 10.1097/01.tp.0000178933.31987.11 16314799

[B89] Merianos D. J. Tiblad E. Santore M. T. Todorow C. A. Laje P. Endo M. (2009). Maternal alloantibodies induce a postnatal immune response that limits engraftment following in utero hematopoietic cell transplantation in mice. J. Clin. Invest 119 (9), 2590–2600. 10.1172/jci38979 19652363 PMC2735937

[B90] Michejda M. Peters S. M. Bacher J. Hernandez L. F. Bellanti J. A. (1992). Intrauterine xenotransplantation of bone marrow stem cells in nonhuman primates. Transplantation 54 (4), 759–762. 1357795

[B91] Mintz B. Anthony K. Litwin S. (1984). Monoclonal derivation of mouse myeloid and lymphoid lineages from totipotent hematopoietic stem cells experimentally engrafted in fetal hosts. Proc. Natl. Acad. Sci. U. S. A. 81 (24), 7835–7839. 10.1073/pnas.81.24.7835 6393129 PMC392247

[B92] Mokhtari S. Colletti E. J. Atala A. Zanjani E. D. Porada C. D. Almeida-Porada G. (2016). Boosting hematopoietic engraftment after in utero transplantation through vascular niche manipulation. Stem Cell Rep. 6 (6), 957–969. 10.1016/j.stemcr.2016.05.009 PMC4912311 27304918

[B93] Moreno R. Martínez-González I. Rosal M. Nadal M. Petriz J. Gratacós E. (2012). Fetal liver-derived mesenchymal stem cell engraftment after allogeneic in utero transplantation into rabbits. Stem Cells Dev. 21 (2), 284–295. 10.1089/scd.2010.0483 21495909 PMC3258433

[B94] Moustafa M. E. Srivastava A. S. Nedelcu E. Donahue J. Gueorguieva I. Shenouda S. S. (2004). Chimerism and tolerance post-in utero transplantation with embryonic stem cells. Transplantation 78 (9), 1274–1282. 10.1097/01.tp.0000137267.17002.b5 15548963

[B95] Muench M. O. Rae J. Bárcena A. Leemhuis T. Farrell J. Humeau L. (2001). Transplantation of a fetus with paternal Thy-1(+)CD34(+)cells for chronic granulomatous disease. Bone Marrow Transpl. 27 (4), 355–364. 10.1038/sj.bmt.1702798 11313664

[B96] Navarro Alvarez N. Zhu A. Arellano R. S. Randolph M. A. Duggan M. Scott Arn J. (2015). Postnatal xenogeneic B-cell tolerance in swine following in utero intraportal antigen exposure. Xenotransplantation 22 (5), 368–378. 10.1111/xen.12186 26314946

[B97] Nguyen Q. H. Witt R. G. Wang B. Eikani C. Shea J. Smith L. K. (2020). Tolerance induction and microglial engraftment after fetal therapy without conditioning in mice with Mucopolysaccharidosis type VII. Sci. Transl. Med. 12 (532). 10.1126/scitranslmed.aay8980 32102934

[B98] Nijagal A. Derderian C. Le T. Jarvis E. Nguyen L. Tang Q. (2013). Direct and indirect antigen presentation lead to deletion of donor-specific T cells after in utero hematopoietic cell transplantation in mice. Blood 121 (22), 4595–4602. 10.1182/blood-2012-10-463174 23610372 PMC3668492

[B99] Nijagal A. Wegorzewska M. Jarvis E. Le T. Tang Q. MacKenzie T. C. (2011a). Maternal T cells limit engraftment after in utero hematopoietic cell transplantation in mice. J. Clin. Invest 121 (2), 582–592. 10.1172/jci44907 21245575 PMC3026737

[B100] Nijagal A. Wegorzewska M. Le T. Tang Q. Mackenzie T. C. (2011b). The maternal immune response inhibits the success of in utero hematopoietic cell transplantation. Chimerism 2 (2), 55–57. 10.4161/chim.2.2.16287 21912720 PMC3166485

[B101] Noia G. Ligato M. S. Cesari E. Visconti D. Fortunato G. Tintoni M. (2008). Source of cell injected is a critical factors for short and long engraftment in xeno-transplantation. Cell Prolif. 41 (1), 41–50. 10.1111/j.1365-2184.2008.00481.x 18181944 PMC6496627

[B102] Noia G. Pierelli L. Bonanno G. Monego G. Perillo A. Rutella S. (2003). A novel route of transplantation of human cord blood stem cells in preimmune fetal sheep: The intracelomic cavity. Stem Cells 21 (6), 638–646. 10.1634/stemcells.21-6-638 14595123

[B103] Omori F. Lutzko C. Abrams-Ogg A. Lau K. Gartley C. Dobson H. (1999). Adoptive transfer of genetically modified human hematopoietic stem cells into preimmune canine fetuses. Exp. Hematol. 27 (2), 242–249. 10.1016/s0301-472x(98)00043-5 10029163

[B104] Orlandi F. Giambona A. Messana F. Marino M. Abate I. Calzolari R. (1996). Evidence of induced non-tolerance in HLA-identical twins with hemoglobinopathy after in utero fetal transplantation. Bone Marrow Transpl. 18 (3), 637–639. 8879630

[B105] Owen R. D. (1945). Immunogenetic consequences of vascular anastomoses between bovine twins. Science 102 (2651), 400–401. 10.1126/science.102.2651.400 17755278

[B106] Palchaudhuri R. Saez B. Hoggatt J. Schajnovitz A. Sykes D. B. Tate T. A. (2016). Non-genotoxic conditioning for hematopoietic stem cell transplantation using a hematopoietic-cell-specific internalizing immunotoxin. Nat. Biotechnol. 34 (7), 738–745. 10.1038/nbt.3584 27272386 PMC5179034

[B107] Palmer E. (2003). Negative selection-clearing out the bad apples from the T-cell repertoire. Nat. Rev. Immunol. 3 (5), 383–391. 10.1038/nri1085 12766760

[B108] Panaroni C. Gioia R. Lupi A. Besio R. Goldstein S. A. Kreider J. (2009). In utero transplantation of adult bone marrow decreases perinatal lethality and rescues the bone phenotype in the knockin murine model for classical, dominant osteogenesis imperfecta. Blood 114 (2), 459–468. 10.1182/blood-2008-12-195859 19414862 PMC2714216

[B109] Peranteau W. H. Endo M. Adibe O. O. Flake A. W. (2007). Evidence for an immune barrier after in utero hematopoietic-cell transplantation. Blood 109 (3), 1331–1333. 10.1182/blood-2006-04-018606 17023584 PMC1785153

[B110] Peranteau W. H. Endo M. Adibe O. O. Merchant A. Zoltick P. W. Flake A. W. (2006). CD26 inhibition enhances allogeneic donor-cell homing and engraftment after in utero hematopoietic-cell transplantation. Blood 108 (13), 4268–4274. 10.1182/blood-2006-04-018986 16954501 PMC1895454

[B111] Peranteau W. H. Hayashi S. Abdulmalik O. Chen Q. Merchant A. Asakura T. (2015). Correction of murine hemoglobinopathies by prenatal tolerance induction and postnatal nonmyeloablative allogeneic BM transplants. Blood 126 (10), 1245–1254. 10.1182/blood-2015-03-636803 26124498 PMC4559936

[B112] Peranteau W. H. Hayashi S. Hsieh M. Shaaban A. F. Flake A. W. (2002). High-level allogeneic chimerism achieved by prenatal tolerance induction and postnatal nonmyeloablative bone marrow transplantation. Blood 100 (6), 2225–2234. 10.1182/blood-2002-01-0166 12200389

[B113] Peranteau W. H. Heaton T. E. Gu Y. C. Volk S. W. Bauer T. R. Alcorn K. (2009). Haploidentical in utero hematopoietic cell transplantation improves phenotype and can induce tolerance for postnatal same-donor transplants in the canine leukocyte adhesion deficiency model. Biol. Blood Marrow Transpl. 15 (3), 293–305. 10.1016/j.bbmt.2008.11.034 PMC2796507 19203720

[B114] Petersen S. M. Gendelman M. Murphy K. M. Torbenson M. Jones R. J. Althaus J. E. (2007). Use of T-cell antibodies for donor dosaging in a canine model of in utero hematopoietic stem cell transplantation. Fetal Diagn Ther. 22 (3), 175–179. 10.1159/000098711 17228153

[B115] Petersen S. M. Gendelman M. Murphy K. M. Torbenson M. Jones R. J. Stetten G. (2013). In utero hematopoietic stem cell transplantation in canines: Exploring the gestational age window of opportunity to maximize engraftment. Fetal Diagn Ther. 33 (2), 116–121. 10.1159/000346211 23343577

[B116] Ramshaw H. S. Crittenden R. B. Dooner M. Peters S. O. Rao S. S. Quesenberry P. J. (1995). High levels of engraftment with a single infusion of bone marrow cells into normal unprepared mice. Biol. Blood Marrow Transpl. 1 (2), 74–80. 9118295

[B117] Rao S. Peters S. Crittenden R. Stewart F. Ramshaw H. Quesenberry P. (1997). Stem cell transplantation in the normal nonmyeloablated host: Relationship between cell dose, schedule, and engraftment. Exp. Hematol. 25 (2), 114–121. 9015211

[B118] Riley J. S. McClain L. E. Stratigis J. D. Coons B. E. Ahn N. J. Li H. (2020). Regulatory T cells promote alloengraftment in a model of late-gestation in utero hematopoietic cell transplantation. Blood Adv. 4 (6), 1102–1114. 10.1182/bloodadvances.2019001208 32203584 PMC7094012

[B119] Riley J. S. McClain L. E. Stratigis J. D. Coons B. E. Li H. Hartman H. A. (2018). Pre-existing maternal antibodies cause rapid prenatal rejection of allotransplants in the mouse model of in utero hematopoietic cell transplantation. J. Immunol. 201 (5), 1549–1557. 10.4049/jimmunol.1800183 30021770 PMC6103848

[B120] Rio P. Martinez-Palacio J. Ramirez A. Bueren J. A. Segovia J. C. (2005). Efficient engraftment of in utero transplanted mice with retrovirally transduced hematopoietic stem cells. Gene Ther. 12 (4), 358–363. 10.1038/sj.gt.3302419 15550924

[B121] Rosler E. S. Brandt J. E. Chute J. Hoffman R. (2000). An *in vivo* competitive repopulation assay for various sources of human hematopoietic stem cells. Blood 96 (10), 3414–3421. 10.1182/blood.v96.10.3414.h8003414_3414_3421 11071636

[B122] Rubin J. P. Cober S. R. Butler P. E. Randolph M. A. Gazelle G. S. Ierino F. L. (2001). Injection of allogeneic bone marrow cells into the portal vein of swine in utero. J. Surg. Res. 95 (2), 188–194. 10.1006/jsre.2000.6044 11162044

[B123] Sagar R. Götherström C. David A. L. Westgren M. (2019). Fetal stem cell transplantation and gene therapy. Best. Pract. Res. Clin. Obstet. Gynaecol. 58, 142–153. 10.1016/j.bpobgyn.2019.02.007 30910447

[B124] Sanna M. Monni G. Ibba R. Argiolu F. Galanello R. Maccioni L. (1999). “In utero stem cell transplantation for beta-thalassemia: A case report,” in Bone marrow transplantation: Stockton Press Houndmills, Basingstoke Rg21 6xs (Hampshire, England), S109.

[B125] Schoeberlein A. Schatt S. Troeger C. Surbek D. Holzgreve W. Hahn S. (2004). Engraftment kinetics of human cord blood and murine fetal liver stem cells following in utero transplantation into immunodeficient mice. Stem Cells Dev. 13 (6), 677–684. 10.1089/scd.2004.13.677 15684835

[B126] Sefrioui H. Donahue J. Srivastava A. S. Gilpin E. Lee T. H. Carrier E. (2002). Alloreactivity following in utero transplantation of cytokine-stimulated hematopoietic stem cells: The role of recipient CD4(-) cells. Exp. Hematol. 30 (6), 617–624. 10.1016/s0301-472x(02)00803-2 12063030

[B127] Shaaban A. F. Kim H. B. Gaur L. Liechty K. W. Flake A. W. (2006). Prenatal transplantation of cytokine-stimulated marrow improves early chimerism in a resistant strain combination but results in poor long-term engraftment. Exp. Hematol. 34 (9), 1278–1287. 10.1016/j.exphem.2006.05.007 16939821 PMC3096442

[B128] Shaaban A. F. Kim H. B. Milner R. Flake A. W. (1999). A kinetic model for the homing and migration of prenatally transplanted marrow. Blood 94 (9), 3251–3257. 10.1182/blood.v94.9.3251.421k10_3251_3257 10556214

[B129] Shangaris P. Loukogeorgakis S. P. Blundell M. P. Petra E. Shaw S. W. Ramachandra D. L. (2018). Long-term hematopoietic engraftment of congenic amniotic fluid stem cells after in utero intraperitoneal transplantation to immune competent mice. Stem Cells Dev. 27 (8), 515–523. 10.1089/scd.2017.0116 29482456 PMC5910037

[B130] Shaw S. W. Blundell M. P. Pipino C. Shangaris P. Maghsoudlou P. Ramachandra D. L. (2015). Sheep CD34+ amniotic fluid cells have hematopoietic potential and engraft after autologous in utero transplantation. Stem Cells 33 (1), 122–132. 10.1002/stem.1839 25186828

[B131] Shaw S. W. Peng S. Y. Liang C. C. Lin T. Y. Cheng P. J. Hsieh T. T. (2021). Prenatal transplantation of human amniotic fluid stem cell could improve clinical outcome of type III spinal muscular atrophy in mice. Sci. Rep. 11 (1), 9158. 10.1038/s41598-021-88559-z 33911155 PMC8080644

[B132] Shields L. E. Gaur L. Delio P. Gough M. Potter J. Sieverkropp A. (2005). The use of CD 34(+) mobilized peripheral blood as a donor cell source does not improve chimerism after in utero hematopoietic stem cell transplantation in non-human primates. J. Med. Primatol. 34 (4), 201–208. 10.1111/j.1600-0684.2005.00110.x 16053498

[B133] Shields L. E. Gaur L. Delio P. Potter J. Sieverkropp A. Andrews R. G. (2004). Fetal immune suppression as adjunctive therapy for in utero hematopoietic stem cell transplantation in nonhuman primates. Stem Cells 22 (5), 759–769. 10.1634/stemcells.22-5-759 15342940

[B134] Shields L. E. Gaur L. K. Gough M. Potter J. Sieverkropp A. Andrews R. G. (2003). In utero hematopoietic stem cell transplantation in nonhuman primates: The role of T cells. Stem Cells 21 (3), 304–314. 10.1634/stemcells.21-3-304 12743325

[B135] Shimoto M. Sugiyama T. Nagasawa T. (2017). Numerous niches for hematopoietic stem cells remain empty during homeostasis. Blood 129 (15), 2124–2131. 10.1182/blood-2016-09-740563 28130213

[B136] Slavin S. Naparstek E. Ziegler M. Lewin A. (1992). Clinical application of intrauterine bone marrow transplantation for treatment of genetic diseases-feasibility studies. Bone Marrow Transpl. 9 (1), 189–190. 1504665

[B137] Srour E. F. Zanjani E. D. Brandt J. E. Leemhuis T. Briddell R. A. Heerema N. A. (1992). Sustained human hematopoiesis in sheep transplanted in utero during early gestation with fractionated adult human bone marrow cells. Blood 79 (6), 1404–1412. 10.1182/blood.v79.6.1404.bloodjournal7961404 1372186

[B138] Srour E. F. Zanjani E. D. Cornetta K. Traycoff C. M. Flake A. W. Hedrick M. (1993). Persistence of human multilineage, self-renewing lymphohematopoietic stem cells in chimeric sheep. Blood 82 (11), 3333–3342. 10.1182/blood.v82.11.3333.3333 7694681

[B139] Stewart F. M. Crittenden R. B. Lowry P. A. Pearson-White S. Quesenberry P. J. (1993). Long-term engraftment of normal and post-5-fluorouracil murine marrow into normal nonmyeloablated mice. Blood 81 (10), 2566–2571. 10.1182/blood.v81.10.2566.bloodjournal81102566 8098231

[B140] Surbek D. V. Holzgreve W. Nicolaides K. H. (2001). Haematopoietic stem cell transplantation and gene therapy in the fetus: Ready for clinical use? Hum. Reprod. Update 7 (1), 85–91. 10.1093/humupd/7.1.085 11212081

[B141] Tai-MacArthur S. Lombardi G. Shangaris P. (2021). The theoretical basis of in utero hematopoietic stem cell transplantation and its use in the treatment of blood disorders. Stem Cells Dev. 30 (2), 49–58. 10.1089/scd.2020.0181 33280478

[B142] Takahama Y. (2006). Journey through the thymus: Stromal guides for T-cell development and selection. Nat. Rev. Immunol. 6 (2), 127–135. 10.1038/nri1781 16491137

[B143] Tanaka Y. Masuda S. Abe T. Hayashi S. Kitano Y. Nagao Y. (2010). Intravascular route is not superior to an intraperitoneal route for in utero transplantation of human hematopoietic stem cells and engraftment in sheep. Transplantation 90 (4), 462–463. 10.1097/TP.0b013e3181eac3c1 20720481

[B144] Tarantal A. F. Goldstein O. Barley F. Cowan M. J. (2000). Transplantation of human peripheral blood stem cells into fetal rhesus monkeys (*Macaca mulatta*). Transplantation 69 (9), 1818–1823. 10.1097/00007890-200005150-00015 10830217

[B145] Taylor P. A. McElmurry R. T. Lees C. J. Harrison D. E. Blazar B. R. (2002). Allogenic fetal liver cells have a distinct competitive engraftment advantage over adult bone marrow cells when infused into fetal as compared with adult severe combined immunodeficient recipients. Blood 99 (5), 1870–1872. 10.1182/blood.v99.5.1870 11861310

[B146] Tondelli B. Blair H. C. Guerrini M. Patrene K. D. Cassani B. Vezzoni P. (2009). Fetal liver cells transplanted in utero rescue the osteopetrotic phenotype in the oc/oc mouse. Am. J. Pathol. 174 (3), 727–735. 10.2353/ajpath.2009.080688 19218349 PMC2665735

[B147] Touraine J. L. Raudrant D. Golfier F. Rebaud A. Sembeil R. Roncarolo M. G. (2004). Reappraisal of in utero stem cell transplantation based on long-term results. Fetal Diagn Ther. 19 (4), 305–312. 10.1159/000077957 15192288

[B148] Touraine J. L. Raudrant D. Laplace S. (1997). Transplantation of hemopoietic cells from the fetal liver to treat patients with congenital diseases postnatally or prenatally. Transpl. Proc. 29 (1-2), 712–713. 10.1016/s0041-1345(96)00432-0 9123493

[B149] Touraine J. L. Raudrant D. Royo C. Rebaud A. Roncarolo M. G. Souillet G. (1989). *In-utero* transplantation of stem cells in bare lymphocyte syndrome. Lancet 1 (8651), 1382. 10.1016/s0140-6736(89)92819-5 2567387

[B150] Troeger C. Perahud I. Moser S. Holzgreve W. (2010). Transplacental traffic after in utero mesenchymal stem cell transplantation. Stem Cells Dev. 19 (9), 1385–1392. 10.1089/scd.2009.0434 20131967

[B151] Turner C. W. Archer D. R. Wong J. Yeager A. M. Fleming W. H. (2015). In utero transplantation of human fetal haemopoietic cells in NOD/SCID mice. Br. J. Haematol. 103 (2), 326–334. 10.1046/j.1365-2141.1998.01003.x 9827901

[B152] Vaags A. K. Gartley C. J. Halling K. B. Dobson H. Zheng Y. Foltz W. D. (2011). Migration of cells from the yolk sac to hematopoietic tissues after in utero transplantation of early and mid gestation canine fetuses. Transplantation 91 (7), 723–730. 10.1097/TP.0b013e31820c85bc 21325997

[B153] Vrecenak J. D. Flake A. W. (2013). In utero hematopoietic cell transplantation-recent progress and the potential for clinical application. Cytotherapy 15 (5), 525–535. 10.1016/j.jcyt.2013.01.003 23415921

[B154] Vrecenak J. D. Partridge E. A. Pearson E. G. Flake A. W. (2020). Simple approach to increase donor hematopoietic stem cell dose and improve engraftment in the murine model of allogeneic in utero hematopoietic cell transplantation. Biol. Blood Marrow Transpl. 26 (1), e21–e24. 10.1016/j.bbmt.2019.08.024 31493540

[B155] Vrecenak J. D. Pearson E. G. Santore M. T. Todorow C. A. Li H. Radu A. (2014). Stable long-term mixed chimerism achieved in a canine model of allogeneic in utero hematopoietic cell transplantation. Blood 124 (12), 1987–1995. 10.1182/blood-2013-11-537571 24869940

[B156] Vrecenak J. D. Pearson E. G. Todorow C. A. Li H. Johnson M. P. Flake A. W. (2018). Preclinical canine model of graft-versus-host disease after in utero hematopoietic cell transplantation. Biol. Blood Marrow Transpl. 24 (9), 1795–1801. 10.1016/j.bbmt.2018.05.020 29802901

[B157] Waldschmidt T. J. Panoskaltsis-Mortari A. McElmurry R. T. Tygrett L. T. Taylor P. A. Blazar B. R. (2002). Abnormal T cell-dependent B-cell responses in SCID mice receiving allogeneic bone marrow in utero. Severe combined immune deficiency. Blood 100 (13), 4557–4564. 10.1182/blood-2002-04-1232 12393436

[B158] Wengler G. S. Lanfranchi A. Frusca T. Verardi R. Neva A. Brugnoni D. (1996). *In-utero* transplantation of parental CD34 haematopoietic progenitor cells in a patient with X-linked severe combined immunodeficiency (SCIDXI). Lancet 348 (9040), 1484–1487. 10.1016/s0140-6736(96)09392-0 8942778

[B159] Wengler G. S. Lombardi G. Frusca T. Alberti D. Albertini A. Parolini O. (2005). In utero transplantation of human cord blood cells into rabbits. Transplantation 80 (2), 282–283. 10.1097/01.tp.0000163503.12780.5e 16041277

[B160] Westgren M. (2006). *In utero* stem cell transplantation. Semin. Reprod. Med. 24 (5), 348–357. 10.1055/s-2006-952156 17123230

[B161] Westgren M. Ringdén O. Bartmann P. Bui T. H. Lindton B. Mattsson J. (2002). Prenatal T-cell reconstitution after in utero transplantation with fetal liver cells in a patient with X-linked severe combined immunodeficiency. Am. J. Obstet. Gynecol. 187 (2), 475–482. 10.1067/mob.2002.123602 12193946

[B162] Westgren M. Ringden O. Eik-Nes S. Ek S. Anvret M. Brubakk A. M. (1996). Lack of evidence of permanent engraftment after in utero fetal stem cell transplantation in congenital hemoglobinopathies. Transplantation 61 (8), 1176–1179. 10.1097/00007890-199604270-00010 8610414

[B163] Witt R. G. Kreger E. M. Buckman L. B. Moradi P. W. Ho P. T. Derderian S. C. (2018a). Systemic multilineage engraftment in mice after in utero transplantation with human hematopoietic stem cells. Blood Adv. 2 (1), 69–74. 10.1182/bloodadvances.2017011585 29344586 PMC5761626

[B164] Witt R. G. Wang B. Nguyen Q. H. Eikani C. Mattis A. N. MacKenzie T. C. (2018b). Depletion of murine fetal hematopoietic stem cells with c-Kit receptor and CD47 blockade improves neonatal engraftment. Blood Adv. 2 (24), 3602–3607. 10.1182/bloodadvances.2018022020 30567724 PMC6306881

[B165] Zanjani E. D. Almeida-Porada G. Ascensao J. L. MacKintosh F. R. Flake A. W. (1997). Transplantation of hematopoietic stem cells in utero. Stem Cells 15 (1), 79–92. discussion 93. 10.1002/stem.5530150812 9368328

[B166] Zanjani E. D. Ascensao J. L. Harrison M. R. Tavassoli M. (1992a). *Ex vivo* incubation with growth factors enhances the engraftment of fetal hematopoietic cells transplanted in sheep fetuses. Blood 79 (11), 3045–3049. 10.1182/blood.v79.11.3045.bloodjournal79113045 1350230

[B167] Zanjani E. D. Ascensao J. L. Tavassoli M. (1993). Liver-derived fetal hematopoietic stem cells selectively and preferentially home to the fetal bone marrow. Blood 81 (2), 399–404. 10.1182/blood.v81.2.399.399 8093667

[B168] Zanjani E. D. Flake A. W. Rice H. Hedrick M. Tavassoli M. (1994). Long-term repopulating ability of xenogeneic transplanted human fetal liver hematopoietic stem cells in sheep. J. Clin. Invest 93 (3), 1051–1055. 10.1172/jci117054 7907601 PMC294034

[B169] Zanjani E. D. Pallavicini M. G. Ascensao J. L. Flake A. W. Langlois R. G. Reitsma M. (1992b). Engraftment and long-term expression of human fetal hemopoietic stem cells in sheep following transplantation in utero. J. Clin. Invest 89 (4), 1178–1188. 10.1172/jci115701 1348253 PMC442977

[B170] Zanjani E. (1994). In utero hematopoietic stem cell transplantation results in donor specific tolerance and facilitates post-natal'boosting'of donor cell levels. Blood 84 (1), 100a.

